# Using Historical Atlas Data to Develop High-Resolution Distribution Models of Freshwater Fishes

**DOI:** 10.1371/journal.pone.0129995

**Published:** 2015-06-15

**Authors:** Jian Huang, Emmanuel A. Frimpong

**Affiliations:** Department of Fish and Wildlife Conservation, Virginia Polytechnic Institute and State University, Blacksburg, Virginia, United States of America; Bournemouth University, UNITED KINGDOM

## Abstract

Understanding the spatial pattern of species distributions is fundamental in biogeography, and conservation and resource management applications. Most species distribution models (SDMs) require or prefer species presence and absence data for adequate estimation of model parameters. However, observations with unreliable or unreported species absences dominate and limit the implementation of SDMs. Presence-only models generally yield less accurate predictions of species distribution, and make it difficult to incorporate spatial autocorrelation. The availability of large amounts of historical presence records for freshwater fishes of the United States provides an opportunity for deriving reliable absences from data reported as presence-only, when sampling was predominantly community-based. In this study, we used boosted regression trees (BRT), logistic regression, and MaxEnt models to assess the performance of a historical metacommunity database with inferred absences, for modeling fish distributions, investigating the effect of model choice and data properties thereby. With models of the distribution of 76 native, non-game fish species of varied traits and rarity attributes in four river basins across the United States, we show that model accuracy depends on data quality (e.g., sample size, location precision), species’ rarity, statistical modeling technique, and consideration of spatial autocorrelation. The cross-validation area under the receiver-operating-characteristic curve (AUC) tended to be high in the spatial presence-absence models at the highest level of resolution for species with large geographic ranges and small local populations. Prevalence affected training but not validation AUC. The key habitat predictors identified and the fish-habitat relationships evaluated through partial dependence plots corroborated most previous studies. The community-based SDM framework broadens our capability to model species distributions by innovatively removing the constraint of lack of species absence data, thus providing a robust prediction of distribution for stream fishes in other regions where historical data exist, and for other taxa (e.g., benthic macroinvertebrates, birds) usually observed by community-based sampling designs.

## Introduction

Understanding species-habitat relationships and the spatial pattern of species distributions is critical in biogeography, biodiversity conservation, and resource management [1, 2]. Through modeling historical ranges, suitable locations could be determined for reintroducing and recovering declining or extirpated species [3, 4]. Based on current biological sampling surveys, species distribution models (SDMs) could be used to design conservation or management plans [5–7]. Conservation managers could predict and mitigate the effect of potential climate and landscape changes on economic or threatened species [8–10], and find strategies to control species invasions [11–13] by updating habitat variables to future scenarios in calibrated models.

One component now limiting the progress of biodiversity conservation and resource management is biological data to support rigorous SDMs [2, 14, 15]. Species occurrence data of high resolution, particularly at large spatial extents, are usually not available or not synthesized into readily usable forms. For example, NatureServe provides the most up-to-date electronic species distribution maps of US freshwater fauna and flora at the HUC-8 (hydrologic unit 8-digit code) level (http://www.natureserve.org/), but neither species-habitat relationships nor subtle temporal shifts in distribution are discernible from maps at such coarse resolutions. This limitation exists largely because gathering occurrence data by sampling each species’ entire habitat range can be time-consuming and costly [14]. Observations of presence for rare, cryptic, and migratory freshwater fishes tend to be particularly spatially sparse, let alone the absence data that ideally require multiple-visit occupancy-based sampling designs. Constrained by data availability, most previous SDMs studies have focused on common or economic species [16, 17], or developed models with only presence observations such as the Maximum-Entropy Species-Distribution Modeling or MaxEnt [18]. However, presence-only models can only estimate realized niche when the assumptions of known prevalence and sampling bias are valid [19], and usually yield less accurate species-habitat associations and species distributions than presence-absence models [14, 20].

Atlases have been the most common approach to present species occurrences at large spatial extents [14]. However, most distribution maps derive data from reports of the occurrence of species (i.e., a snapshot of presences), thus they only provide limited information on species abundances and relative habitat suitability. It is easy to underestimate presence consistently in interpreting these maps, because a species is considered absent in locations subjected to no or very low sampling effort. Particularly, non-game species of fish that have not been the focus of any specific conservation studies and species whose detection depend strongly on sampling gear, effort, or habitat type will tend to show higher numbers of false absences. Alternatively, researchers have used museum records to evaluate species distribution across multiple states or such large sampling units. Yet, some common limitations of museum data have been identified, including: 1) they may not accurately locate the position of records collected before the era of GPS [21], 2) they are usually collected with varied sampling approaches and intensities, 3) they span long time periods in which the habitats might have changed substantially, 4) and they are not sufficient in quantity to delineate full distribution ranges of species and develop robust models [13]. These aspects of sampling biases tend to inflate false negative or positive rates in the less sampled areas, and underestimate species’ dispersal and invasion ability in prediction studies [1, 21, 22]. A framework that can appropriately synthesize species occurrence from field surveys and literature would provide an avenue to fill the gaps in data for modeling and predicting the spatial distribution of species.

We propose a framework for modeling species distributions using historical presences of species recorded in high-resolution atlases and absences inferred from locations where historical presences have been recorded for other species known to be typically sampled as part of a community. Applying the framework to freshwater fishes of the United States, non-game species are better indicators of community sampling. Unlike non-game species, the presence of game species in a sample can be of questionable utility in inferring habitat suitability because populations of game species exist in many suboptimal habitats due to repeated stocking. In addition, whereas game species tend to be targeted for recreation and oversampled, non-game species appear in presence records predominantly as part of community samples. Accumulated over many years, we propose that such samples offer a strong evidence of absence where a species has never been observed but presence records of other species exist (S1 File, Figure A in S3 File).

Species observed over multiple spatial and temporal scales in a defined geographic area belong to a metacommunity [23–25]. In practice, developing such a metacommunity sample involves collating historical occurrences of fish species from different sources and deriving absences for one species from known presences of other species. Communities in a defined metacommunity are assumed spatially connected by migrating and dispersing individuals and species [23, 24], and local community compositions are determined by the regional species pool and regulated by local environmental factors according to two of the prevailing perspectives of metacommunities [25, 26]. Whereas species present in a sampling unit belong to the same regional pool, they may not all have co-existed in that unit at any point in time, and coexisting species may not be observed in a single sampling visit either, due to the variability in sampling technique, timing, effort, and detection rate of different species [27–30]. The temporal and spatial dependencies of occurrence are particularly strong for vagile species, such as fish, which regularly move among feeding, breeding, and over-wintering or summer habitats [31]. The compilation and documentation of the metacommunity database are provided in the S1 File.

In this study, we used boosted regression trees (BRT), logistic regression and MaxEnt models to assess the performance of a historical metacommunity database, with the overarching objective of 1) comparing presence-only and presence-absence models, where we infer the absence of a species from accumulated evidence of the presence of other fish species. Additionally, we investigated the effect of 2) data resolution at two levels (i.e., the National Hydrography Dataset-NHD segment level and Hydrologic Unit Code-HUC12 level), 3) species’ rarity and sampling prevalence, and 4) spatial autocorrelation on model performance. We modeled habitat suitability and distribution of 76 selected freshwater fish species (representing approximately 10% of described freshwater fish species of the United States) exhibiting a range of rarity in four basins of the United States. We used principal coordinate analysis of neighbor matrices, PCNM [32], to incorporate spatial autocorrelation into the species distribution models as a means to evaluate the effects of spatial autocorrelation on model performance. We assessed specific habitat requirements for the selected species through partial dependence plots derived from the BRT. Data resolution, species’ rarity and sampling prevalence, and spatial autocorrelation, are major known but not fully understood factors affecting the behavior and performance of SDMs, and likely to corrupt inference if not properly controlled in the quest to investigate any major hypothesis. Our main hypothesis was that presence-absence models developed with inferred absences would outperform presence-only models. If this hypothesis is found to be valid, then the existence of vast historical freshwater fish presences for the entire United States, synthesized into a single metacommunity database, constitutes an enormous resource for SDMs to help address myriad ecological, conservation, and resource management problems.

## Materials and Methods

### Selecting study basins and fish species

**Fig 1:**
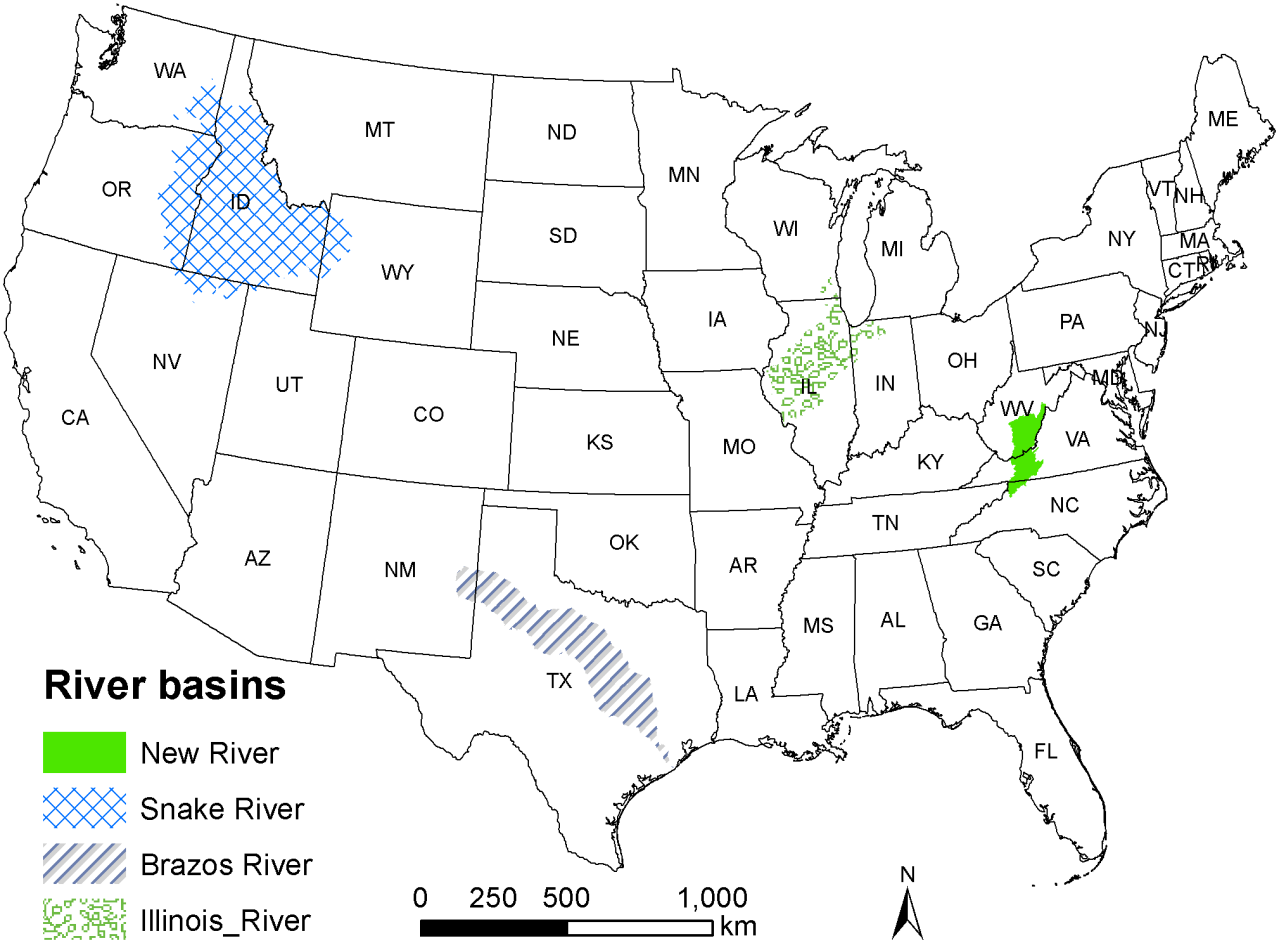
A map showing the distribution of four river basins (i.e., New River, Illinois River, Brazos River, and Snake River) selected for this study in the contiguous United States. We can see that all these four rivers pass through multiple states. Fish presence data are sufficient in these four basins in the *IchthyMap* database for developing and validating species distribution models (S2 File). Specifically, the number of presence records of non-game species used to develop species distribution models was 2,716 for Brazos River Basin, 5,635 for Illinois River Basin, 5,192 for New River Basin and, 412 for the Snake river Basin.

It is imperative for the evaluation of a database and for comparison of different models, to include a variety of regions and a range of common and rare species so that limitations of the proposed modeling approach can be uncovered and explicated. We selected four basins in the United States for this study: New River, Illinois River, Brazos River, and Snake River, meeting criteria of data availability and geographic diversity (Fig 1). The four selected river basins spanned a range of climate, physiography, and anthropogenic influences (e.g., hydrological alterations, agriculture, and urbanization). The Brazos River is warmer than other three basins and it showed narrower range and smaller variation in temperature [33]. The dominant landscape in the New River, Illinois River, Brazos River, and Snake River basins were forest/agriculture, agriculture/urban, forest/grassland, and grassland/forest, respectively. We selected 76 fish species (Table A in S3 File) with different rarity and distributional characteristics from the four river basins to develop habitat suitability and species distribution models. The 76 freshwater species belong to 15 families, and together represent approximately 10% of all currently described freshwater fish species of the United States and a phylogenetically diverse subset of species. The attributes considered in the species selection included a variety of macrohabitat preferences, body size, migration ability, and temperature tolerances [34] and the three common dimensions of rarity—range size, habitat breadth, and local population size [35, 36].

We used the inter-confluence segments of the enhanced 1:100,000 resolution National Hydrographic Dataset (NHDplusV2) as the primary study units. NHDplusV2 is a geographic and hydrologic framework dataset that has been widely applied to the environmental assessment and stream habitat management by the US Environmental Protection Agency (USEPA), US Geological Survey (USGS), and other agencies. Matching the NHDplusV2 resolution (1:100K) allowed for convenient retrieval of numerous environmental (habitat) variables organized by stream segments and network accumulated attributes and for predicting species distribution at high resolution. We also coarsened the habitat and fish occurrence data to HUC12 (12-digit hydrologic unit code) level to examine the effect of data resolution on model performance, and more comprehensively compare different modeling approaches (i.e., presence-absence model versus presence-only model at both NHD segment and HUC12 level).

### Developing species distribution models

The species distribution models we developed in this study are habitat suitability models.

We used the definitions of Kearney [37] for environment-“the biotic and abiotic phenomena surrounding and potentially interacting with an organism” and for habitat-“a description of a physical place, at a particular scale of space and time, where an organism either actually or potentially lives”. Among over 50 available statistical approaches for SDMs, we selected to compare logistic regression under the Lasso (least absolute shrinkage and selection operator) regularization [38], boosted regression tree (BRT) model [39] and the Maximum-Entropy, MaxEnt [18, 40]. Logistic regression has been conventionally used in SDM studies [8, 41]; using Lasso allows mitigation of multicollinearity, and selection of an optimal set of predictor variables. BRT is a more recent machine-learning approach that has outperformed counterparts in few comparative studies and reviews [1, 2]. MaxEnt was found superior to other presence-only models (e.g., GARP and bioclim) in previous comparative studies [1].

In logistic regression, the probabilities of a defined success (e.g., presence of species at a site in this study) can be modeled with a set of the predictor variables, using a logistic link function as follows:

$$log\frac{p(y_{i}=1|x_{i})}{p(y_{i}=0|x_{i})}=\sum_{j=0}^{k}\beta_{j}x_{ij}$$ (1)

Where *p*(*y*_*i*_ = 1|*x*_*i*_) is the probability of presence at site *i*, *y*_*i*_ is species presence (1) or absence (0), *x*_*i*_ is a vector for values of predictor variables, and *β’*s are regression coefficients. The coefficients are usually estimated by optimizing the likelihood function:

$$L\left(\beta \right)=p\left(\beta \text{|}Data\right)=\prod_{i=1}^{n}p{\left(x_{i}\right)}^{y_{i}}{\left(1-p\left(x_{i}\right)\right)}^{1-y_{i}}$$ (2)

Under the Lasso regularization, the objective function is:

$$\text{log}\left(L\left(\beta \right)\right)+\lambda \sum_{j=0}^{k}\left|\;\beta_{j}\right|$$ (3)

where $\sum_{j=0}^{k}\left|\;\beta_{j}\right|\leq S$ is the constraint added on the maximum likelihood optimization, and *λ* is the regularization or penalty parameter that needs to be tuned through validation. We implemented the Lasso-version logistic models in the R statistical program [42] with the package ‘glmnet’ [43].

Boosted regression trees (BRT) developed by Friedman et al. [39] have gained popularity in recent studies of species distribution models. Boosting is the algorithm that ensembles individual classifiers (e.g., classification trees, regression trees) and sequentially fine-tunes the model by using weighted average of predictions [39]. The optimal number of trees were determined through minimizing the loss function in terms of deviance reduction, while achieving a good balance between tree complexity and learning rate [1]. Most currently used BRT models also incorporate bagging algorithms. Bagging strategies (i.e., both samples and predictors are randomly sub-sampled without replacement from the full dataset) are applied at each iteration to control overfitting (by bagging samples) and incorporate complex non-linear relationships (by bagging predictors) [44]. Analogous to other tree-based models, BRT models do not require pre-selecting or re-scaling predictor variables; instead, contribution (or importance) of each predictor variable are calculated based on the frequency of a variable being selected for splitting, weighted by the squared improvement to the model from each split across all trees [45]. Other appealing features of BRT models include resistance to outliers and multicollinearity, and applicability to data of small sample size but many predictors (i.e., the n<<p problem) [1, 39]. We implemented the boosted regression tree models with the R package ‘dismo’ [46]. We evaluated the performance of logistic and BRT models in terms of AUC (i.e., the area under the receiver operating characteristic (ROC) curve) in both training and 5-fold cross-validation processes. An ROC curve is a plot of sensitivity (true positive rate) against 1− specificity (true negative rate) at varying discrimination thresholds. The area under the ROC curve (AUC) ranging from 0 to 1 measures the average diagnostic accuracy across various threshold settings on the probability of presence [47]. In the initial analyses, we found other performance measures (e.g., correlations of observed/predicted species occurrence, and deviance) to be significantly correlated with AUC, so for brevity only AUC is shown in the results.

MaxEnt is specialized from the statistical mechanics theories for species distribution model with only presence data [40]. Entropy maximizes as the system disperses to equilibrium over time [48]. The distribution of maximum entropy is most spread out, and equivalent to the uniform distribution [18]. From the ecological perspective, MaxEnt essentially searches the probability distributions of maximum entropy that satisfies all constraints (i.e., the expectation of each environmental variable conditional on species presence needs to match its sampled mean). Environmental variables that have sound ecological basis generally impose strong constraints, which serves as a criterion to measure variable importance and variable selection in MaxEnt. As a generative machine learning approach, MaxEnt could fit complex species-habitat relationships and incorporate multiple types of predictors and interactions thereof. MaxEnt [18] has been developed as a shareware that can be downloaded from www.cs.princeton.edu/~schapire/maxent/. We used the inferred absences from the metacommunity matrices for the MaxEnt absences, instead of pseudo absences randomly drawn from the background (a default setting in the MaxEnt). This change in setting should lower the false negative error rate and make the AUC from MaxEnt models comparable to the other models.

The habitat factors considered in this study were in seven categories: climate, geology, hydrology, stream morphology, land use/land cover, disturbance, and water chemistry (Table 1). The climate data (e.g., temperature, precipitation) were obtained from the PRISM climate group [33]. The land cover data in 1980’s for each NHD inter-confluence catchment and HUC12 watershed were derived from the USGS Land Cover Institute [49]. Other environmental variables of biological importance to stream fish identified in the literature were retrieved from NHDplusV1 and NHDplusV2 [50–52]. In addition, we obtained the habitat quality score from the National Fish Habitat Action Plan (NFHAP) databases [53]. For each set of highly correlated variables (Pearson’s |*r*| > 0.8), only one was kept to minimize multicollinearity. We examined the species-habitat relationships with the partial dependence plots of the optimized boosted regression model for each species.

**Table 1:** The sources and descriptions of environmental variables used to develop species distribution models for the 76 native stream fish species in the United States.

Variable	Type	Source	Description
**COMID**	/	NHDplusV2	Common identifier of an NHD flow line
**SINU**	Stream morphology	NHDplusV2	Sinuosity. Reach length divided by straight line length (straight line from beginning node to end node of reach)
**ELE**	Geology	NHDplusV2	Mean elevation in meters
**SLP**	Geology	NHDplusV2	Mean slope in degrees
**RDX**	Disturbance	NHDplusV2	Number of road-stream crossings per inter-confluence catchment
**BFI**	Hydrology	NHDplusV2	The ratio of base flow (i.e., the component of streamflow attributed to ground-water discharge) to total flow, expressed as a percentage
**SO**	Stream morphology	NHDplusV2	Stream order [54]
**DRA**	Stream morphology	NHDplusV2	Total area of catchment (Square meters)
**MFU**	Hydrology	NHDplusV2	Mean Annual Flow in cubic feet per second (cfs) at bottom of flowline as computed by Unit Runoff Method
**MVU**	Hydrology	NHDplusV2	Mean Annual Velocity (fps) at bottom of flowline as computed by Jobson [55]
**FHS**	Disturbance	NFHAP	An index of cumulative disturbance of catchments of inter-confluence stream segments^a^
**NT**	Water chemistry	NHDplusV1	Sum total of Nitrogen in the catchment in kilograms
**PT**	Water chemistry	NHDplusV1	Sum total of Phosphorus in the catchment in kilograms
**POP**	Disturbance	NHDplusV1	Human population density (Persons per square kilometer multiplied by 10)
**TMI**	Climate	PRISM	20-Year (1961–1980) average annual minimum temperature in Celsius multiplied by 100 for each NHDplus catchment
**TMA**	Climate	PRISM	20-Year (1961–1980) average annual maximum temperature in Celsius multiplied by 100 for each NHDplus catchment
**TM**	Climate	PRISM	20-Year (1961–1980) average temperature in Celsius multiplied by 100 for each NHDplus catchment
**PPT**	Climate	PRISM	20-year (1961–1980) average annual precipitation in millimeters multiplied by 100 (Millimeters multiplied by 100)
**C_UB**	Land use/land cover	USGS-LCI	percentage of urban in the inter-confluence catchment
**C_AG**	Land use/land cover	USGS-LCI	percentage of agriculture in the inter-confluence catchment
**C_FR**	Land use/land cover	USGS-LCI	percentage of forest in the inter-confluence catchment
**C_WT**	Land use/land cover	USGS-LCI	percentage of water in the inter-confluence catchment
**D_AG**	Land use/land cover	USGS-LCI	percentage of agriculture in the HUC-12 watershed
**D_FR**	Land use/land cover	USGS-LCI	percentage of forest in the HUC-12 watershed
**D_WT**	Land use/land cover	USGS-LCI	percentage of water in the HUC-12 watershed
**D_UB**	Land use/land cover	USGS-LCI	percentage of urban in the HUC-12 watershed

Data are from NHDplusV1 [50] and NHDplusV2 [51], NFHAP [53], USGS-LCI [49], and PRISM [33]. The environmental variables, if not specified, were measured per inter-confluence river segment.

^a^ This index is calculated based on 15 disturbance variables [6]. The influence of each distribution variable was weighted by the results of multiple linear regression of all variables against a commonly used biological indicator of habitat condition (i.e., percent intolerant fishes at a site).

We tested whether incorporating spatial autocorrelation would improve the performance of the species distribution models, using the principal coordinate analysis of neighbor matrices (PCNM) approach [32, 56] in the R package ‘PCNM’ [57]. In the PCNM procedure, we first created a Euclidean distance matrix among all sampled stream segments in each of the four basins. We then truncated the distance matrices to a lower triangular matrix (i.e., elements above the diagonal are set to 0). Mutually orthogonal eigenvectors were then extracted from the truncated matrix, and those spatial eigenvectors associated with positive eigenvalues and significant Moran’s I were kept to form the spatial matrix. Moran’s I [58] measures spatial autocorrelation based on both the values and locations of a variable. The null hypothesis in the Moran’s I test is that there is no spatial autocorrelation in the tested variable. This null hypothesis is rejected if there is a strong clustered or dispersed pattern in the tested variable. The decision for the Moran’s I test is usually based on the *p-*value calculated by a permutation on the values of the tested variable among the study units, or by approximating the Moran’s I value to normal score. To incorporate the spatial information into the environmental predictors, we built multivariate regression models with environmental variables as responses and spatial matrix as predictors [59]. We then used the predicted (i.e., ‘spatialized’) values of the environmental variables from the multivariate regression as the model matrix instead of the raw environmental matrix in the spatial models.

After developing species distribution models with procedures described above, we used ANCOVA [60] to examine the effect of model choice, data resolution, species’ rarity, and spatial autocorrelation on AUC, with species and basin as blocking factors and family number as a covariate. The effects of species and basins were blocked because only models from the same dataset (species × basin) are comparable. Phylogenetic relationships among the species we studied might be another source of non-independence, so we used the family number [61] as a surrogate of the phylogenetic eigenvector and treated it as a covariate in the ANCOVA [62]. We used the Box-Cox transformation [63] on the AUC to ensure that the linear model assumptions of normality of residuals and constant variance were valid.

We further selected two species in the rare species group (Candy darter, *Etheostoma osburni* and Spotfin shiner, *Cyprinella spiloptera*) and two species in the common species group (Bigmouth chub, *Nocomis platyrhynchus* and Northern hog sucker, *Hypentelium nigricans*) to examine the effect of prevalence on SDM performance under training and validation. The observed prevalence (i.e., the proportion of presences among all the observations in the raw data) of *E*. *osburni* and *C*. *spiloptera* were little higher than 0.1; logistic regression models could not converge and cross-validation was not feasible for species with lower prevalence. For the two rare species, we kept the total sample size (i.e., the sum of presence and absence records randomly sampled) at 100 while varying the proportion of presences between 0.1 and 0.9. For the two common species (*N*. *platyrhynchus* and *H*. *nigricans*) we first set the total sample size at 300, and decreased to 100 to evaluate the effect of sample size, in addition to the effect of prevalence. We varied prevalence by randomly sampling different ratios of presence and absence records without replacement. For example, we randomly sampled 10 observations from presences and 90 observations from absences to generate prevalence of 0.1, giving a sample size of 100. We built logistic model for each sample and calculated the AUC in the fitting and 10-fold cross validation. We applied a bootstrapping resampling procedure to obtain the mean AUC values over 100 models for each setting of species and prevalence.

## Results

### Summary of model performance

A total of 13,955 fish presence records occurring on 1,933 NHDplusV2 segments were used to produce species distribution models for the 76 species in the four basins (Fig 1). The choice of model and species’ rarity designation were the factors that significantly affected the model performance in terms of validation AUC at alpha = 0.05, according to the ANCOVA (Table 2). Additionally, spatial autocorrelation significantly affected model performance at alpha = 0.1 level.

**Table 2:** A summary on the Analysis of covariance, ANCOVA [58].

Source	D.F.	M.S.	F	*p*-value
**Treatment factors**				
Model type	2	0.016	86.291	< 0.001
Spatial	1	0.00079	3.954	0.0504
Rarity	7	0.000936	5.012	< 0.001
Resolution	1	0.000366	1.957	0.163
**Block factors and Covariate**				
Basin	3	0.0004	2.153	0.093
Species	75	0.00083	4.463	0.035
Family number (covariate)	1	0.000017	0.090	0.764
**Residuals**	420	0.000187		

ANCOVA was used to evaluate the effect of model types, incorporation of spatial autocorrelation, species’ rarity type, and data resolution on the performance of species distribution models in terms of the area under the receiver operating characteristic (ROC) curve (AUC). Degree of freedom (D.F.), mean square (M.S.), F statistic and *p*-value are listed in this table.

### The effect of model choice

**Fig 2:**
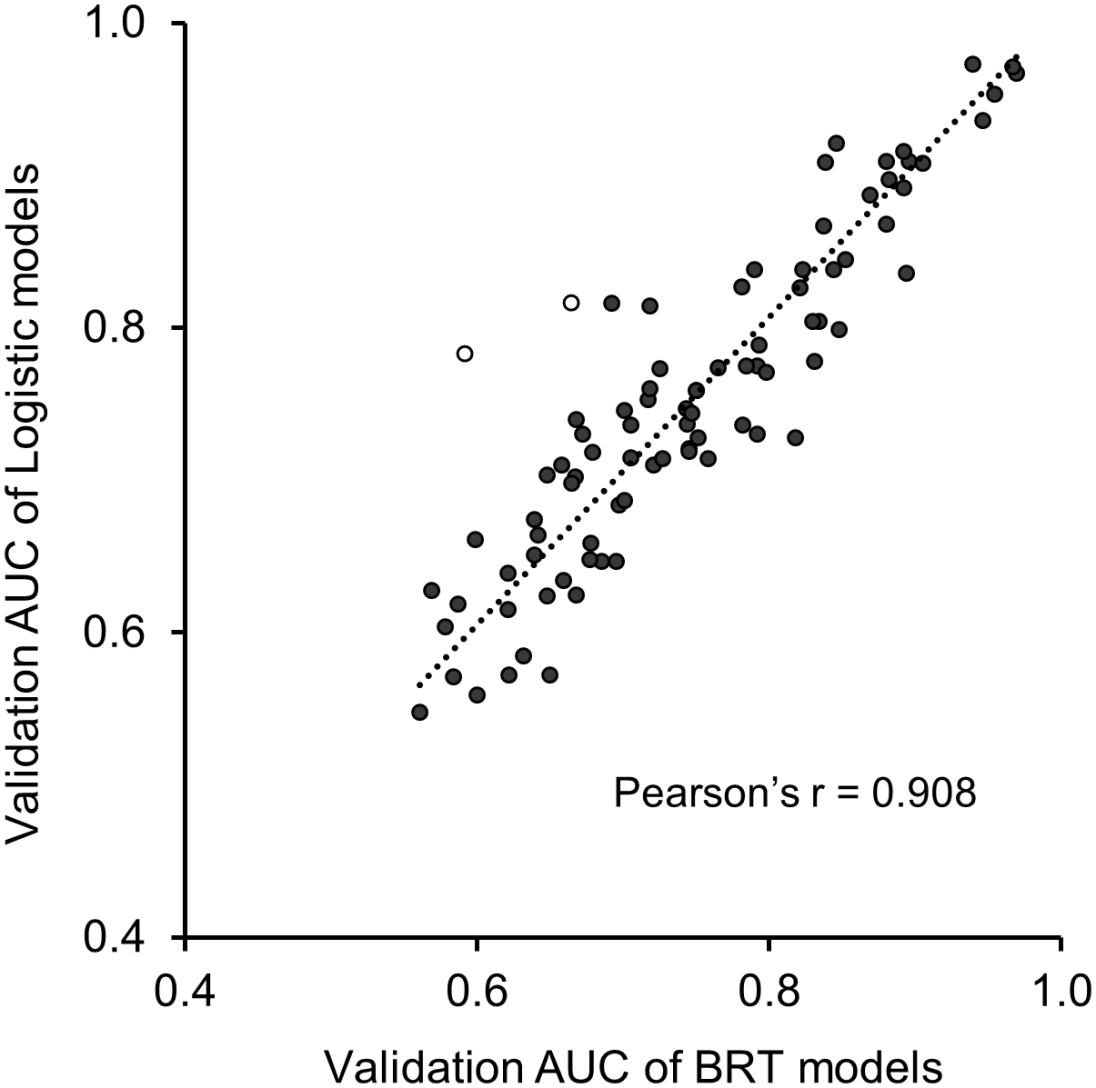
Comparing the performance of Lasso logistic regression model and boosted regression tree (BRT) models in terms of the area under the receiver-operating-characteristic (ROC) curve in the 5-fold cross validation for 76 species in the four selected river basins (i.e., New River, Illinois River, Brazos River and Snake River). The results from the two set of models were generally in agreement, with Pearson’s *r* over 0.9. For fish species Mountain whitefish, *Prosopium williamsoni* and Torrent sculpin, *Cottus rhotheus* (marked as circles) in the Snake River where occurrence data was relatively sparse, the Lasso logistic models outperformed the BRT models.

The presence-absence model, Lasso logistic regression, outperformed the presence-only model, MaxEnt, in the 5-fold cross validation for the 76 study fish species (Table 3). In spite of the vast difference in training performance, validation AUC was not different between the two presence-absence models, Lasso logistic model and BRT, according to the post hoc group comparisons in the Tukey's test [64] (Table 3). The correlation between validation AUC of BRT and validation AUC of logistic models was very high, with Pearson’s correlation over 0.90 (Fig 2). We focused on analyzing BRT models for brevity since the performance of the logistic model agreed in terms of the validation AUC (Fig 2, Table B in S3 File). In addition, the BRT model provided a richer output for model interpretation, in the form of partial dependence plots and variable importance rankings.

**Table 3:** A table summarizing the Tukey's test [6 4] after the analysis of variance that evaluated the sources of effects on the performance of species distribution models.

Treatments compared	Difference	Lower bound	Upper bound	*p*-value
**Model types**				
LM-BRT	-0.010	-0.034	0.014	0.579
MaxEnt-BRT	-0.136	-0.165	-0.107	< 0.001
MaxEnt-LM	-0.126	-0.154	-0.098	< 0.001
**Incorporation of spatial autocorrelation**				
Yes-No	0.020	0.001	0.038	0.037
**Data resolution**				
NHD-HUC	0.017	-0.005	0.038	0.123
**Rarity types**				
B-A	0.092	0.039	0.146	< 0.001
C-A	0.038	-0.002	0.079	0.074
D-A	0.031	-0.043	0.106	0.905
E-A	0.021	-0.028	0.069	0.901
F-A	0.026	-0.043	0.095	0.945
G-A	-0.009	-0.126	0.108	1.000
H-A	-0.010	-0.079	0.059	1.000
C-B	-0.054	-0.116	0.008	0.140
D-B	-0.061	-0.149	0.027	0.408
E-B	-0.072	-0.139	-0.004	0.029
F-B	-0.066	-0.150	0.017	0.239
G-B	-0.101	-0.227	0.024	0.219
H-B	-0.102	-0.185	-0.018	0.005
D-C	-0.007	-0.088	0.073	1.000
E-C	-0.018	-0.075	0.040	0.982
F-C	-0.012	-0.088	0.064	1.000
G-C	-0.048	-0.169	0.073	0.932
H-C	-0.048	-0.124	0.028	0.529
E-D	-0.011	-0.096	0.074	1.000
F-D	-0.005	-0.103	0.093	1.000
G-D	-0.041	-0.177	0.096	0.985
H-D	-0.041	-0.139	0.057	0.909
F-E	0.006	-0.075	0.086	1.000
G-E	-0.030	-0.154	0.094	0.996
H-E	-0.030	-0.111	0.050	0.946
G-F	-0.035	-0.169	0.098	0.993
H-F	-0.036	-0.130	0.058	0.943
H-G	0.000	-0.134	0.133	1.000

The three model types compared are logistic model (LM), boosted regression trees (BRT), and MaxEnt models. The descriptions of the rarity types A-H are provided in Table A in S3 File.

### The effect of species’ rarity and prevalence

Model accuracy was slightly higher for rare species as defined by Pritt and Frimpong [36]. Particularly, species in the rarity Type B and Type D had AUC over 0.75 in the BRT cross validation, which outperformed most species in other rarity types (Table 3). Cross-validation AUC of models for species in the rarity B and C was significantly higher than AUC for species in the rarity A, according to the post hoc group comparisons. Rarity Type B, C and D are species with large geographic ranges but small local populations [35, 36].

**Fig 3:**
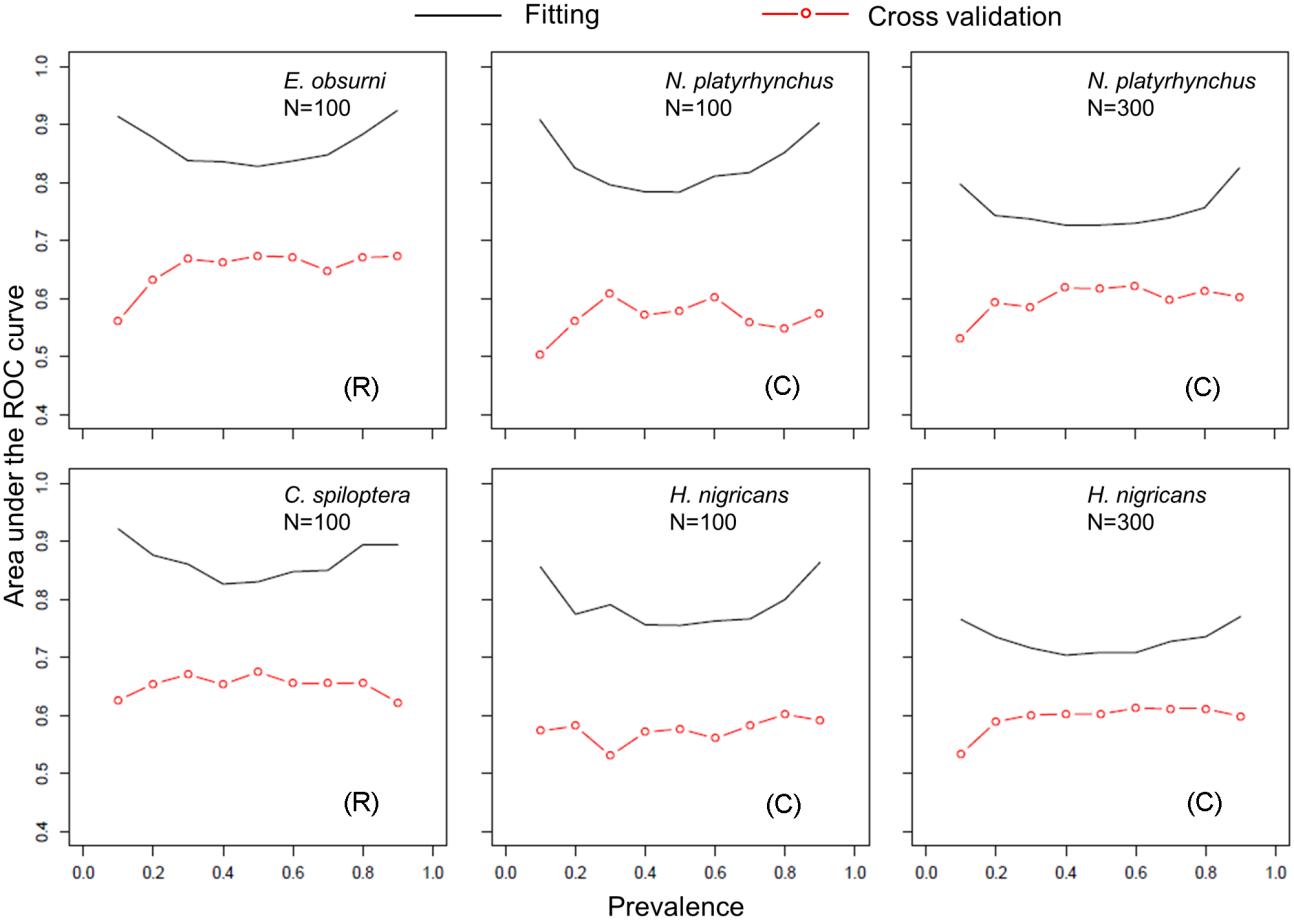
The effect of prevalence (i.e., the proportion of presences among all the observations) on the performance of species distribution models. The total sample size (N) for the two rare species (R), Candy darter (*Etheostoma osburni*) and Spotfin shiner (*Cyprinella spiloptera*), was set at 100; while N was decreased from 300 to 100 for the two common species (C), Bigmouth chub (*Nocomis platyrhynchus*) and Northern hog sucker (*Hypentelium nigricans*), to evaluate the effect of sample size.

The training AUC in the logistic model exhibited a U-shaped response to prevalence for both rare and common species (Fig 3). The total sample size (N) seemed to negatively affect model fitting since models with N = 100 had higher AUC than models with N = 300 in the fitting, for the two species examined with varying sample sizes. In contrast, the U-shaped response of AUC to prevalence disappeared in the 10-fold cross validation; and decreasing the total sample size for common species did not result in increased AUC in the cross validation. The cross-validation AUC of the BRT models had a negative nonlinear relationship with the observed prevalence (i.e., the proportion of presences among all the observations) of the species, indicating that habitat suitability is easier to quantify when variance in occurrence is low (Figure B in S3 File).

### Spatial versus non-spatial models

The ANCOVA (Table 2) showed that incorporating spatial autocorrelation improved model performance in terms of cross-validation, with *p*-value = 0.0504. AUC Specifically, model accuracy increased conspicuously in the spatial models for Yellow bullhead (*Ameiurus natalis*), Orangespotted sunfish (*Lepomis humilis*) and Longnose gar (*Lepisosteus osseus*) in the Brazos River, and Shorthead sculpin (*Cottus confusus*) in the Snake River. For instance, the Moran’s I test [56] on deviance residuals became non-significant (*p*-value > 0.05) after accounting for spatial autocorrelation in the logistic models for Longnose gar (*L*. *osseus*) in the Brazos River.

### Species-habitat relationships

**Fig 4:**
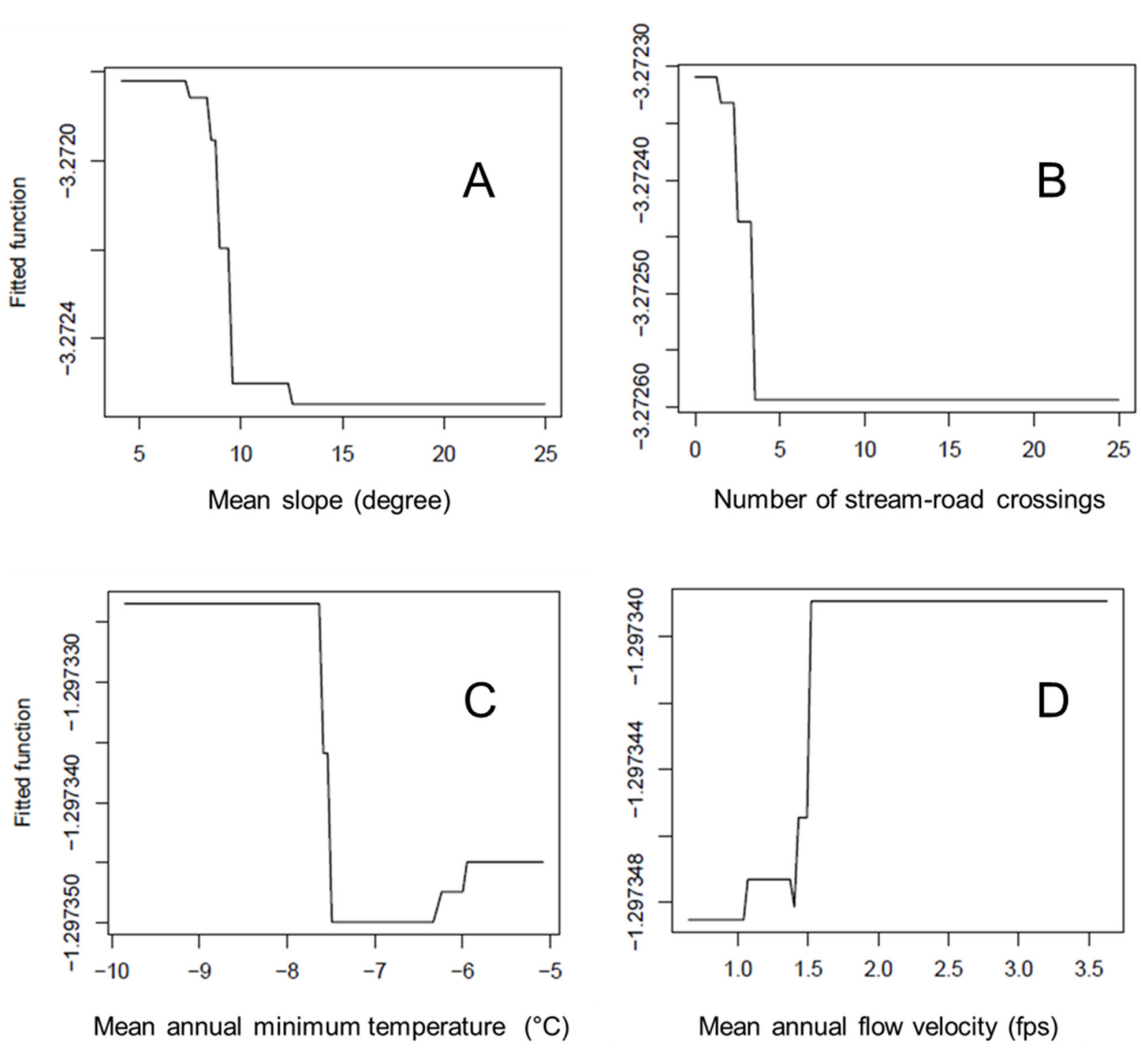
Examples of using partial dependence curves to capture ecological thresholds of spatial distribution of species. For example, the thresholds of mean slope (degree) in the watershed and number of stream-road crossings were identified for Rainbow darter (*Etheostoma caeruleum*) in the panel A and B. The thresholds of 20-year (1961–1980) average annual minimum temperature and mean annual flow velocity were identified for Mountain redbelly dace (*Chrosomus oreas*) in the panel C and D.

We examined species-habitat relationship using measures of variable importance (or contribution) and partial dependence plots in the BRT models (Table C in S3 File). In the non-spatial models, Base flow index (BFI), elevation (ELE), mean annual in-stream flow (MFU), 20-year average minimum January temperature (TMI), 20-year average maximum July temperature (TMA), percentage of agriculture in the watershed (D_AG), annual precipitation (PPT), human population density (POP), drainage area (DRA), and fish habitat score (FHS) were the 10 most important predictors among the 25 environmental variables examined. Generally, these variables related to fish occurrence non-linearly, including polynomial forms, and sudden change after thresholds were common (Fig 4). The hydrology-related variables (e.g., BFI, MFU and PPT) were positively related to the occurrence of most fish species, but constant high flows or floods could be negative force for some species, particularly those living in steep mountain streams in the New River basin, such as Candy darter (*Etheostoma osburni*), Longnose dace (*Hypentelium nigricans*) and Rosyface shiner (*Notropis rubellus*). Temperature, particularly extreme weather events in the winter and summer, were important factors constraining spatial distributions of most fish species, except for those living in the Brazos River basin. Majority of species responded negatively to habitat degradation, indicated by their associations with the fish habitat score. Nevertheless, tolerant and frequently introduced bait species such as Fathead minnow *(Pimephales promelas*) and Common shiner (*Luxilus cornutus*) appeared to be favored in habitats with intense human activities (e.g., high population density and high road density).

The 10 key predictor variables identified in the non-spatial BRT models remained in the spatial BRT models, but the most important predictor changed for 46 out of 86 models (Table D in S3 File). The rank and percent contribution of the top three variables in the non-spatial models, base flow index (BFI), measures of temperature and elevation, dropped in 68%, 72% and 81% spatial models respectively (Table D in S3 File), suggesting that the importance of these variables may be inflated in the non-spatial models due to their built-in spatial dependence. Meanwhile, measures of local stream catchment disturbance, such as fish habitat score and land use type, gained more weights in the spatial models.

## Discussion

We have successfully demonstrated the utility of a high-resolution metacommunity database developed by integrating historical freshwater fish occurrences from state and national atlases and databases for modeling species distributions. We have also shown that at the highest resolution, where such comprehensive datasets are most difficult to come by, presence-absence models outperform presence-only models in the critical step of model validation. Our results corroborate other studies that have previously suggested that the performance of a species distribution model depends on: 1) the data quality [65, 66], 2) choice of statistical modeling technique [2, 22, 67], 3) species’ traits [68, 69], and 4) incorporation of spatial autocorrelation [54, 70–72]. We went further to show how these factors specifically affect models. The proposed framework of collating accumulated high-resolution species presence records into a metacommunity database, including inferred absence of species will serve as a comprehensive tool for understanding species-habitat relationships at multiple spatial scales and help improve conservation and management of taxa. We suggest using the inferred absences based on the metacommunity data rather than pseudo absences randomly sampled from the background in the MaxEnt presence-only models. This adjustment could mitigate the false negative errors in the prediction and make the MaxEnt models comparable with the presence-absence models.

Presence observations were collated despite the different sampling techniques and crews, and more importantly, absences could be inferred from locations where historical presences have been recorded for other species, as long as there is no reason to conclude that sampling overwhelmingly targeted particular species. The approach used in inferring absences has a theoretical root in Bayesian reasoning [19] and these absences are presumed to be more accurate than the pseudo-absences that are randomly sampled from the background in the study area by default a presence-only model such as MaxEnt. The accuracy of inferred absences will be influenced by the resolution of species distribution atlases, GIS procedures (e.g., geo-referencing, snapping) and the accuracy of the habitat template to which the scanned presences are ultimately transposed. Including absences enable presence-absence models to accurately estimate the realized niche of a species [19] and spatial autocorrelation can also be conveniently incorporated into these distribution models. Considering the financial cost, limited time, and the risk of sampling certain rare and vulnerable species to extinction, better utilization should be made of the data that have been gathered by researchers and government agencies through investments made over many decades. Such efforts would thus particularly facilitate delineating habitats for rare or endangered species, the conservation planning for which has been often constrained by data availability. As an illustration, relatively good model performance and accurate species-habitat relationship were obtained without new sampling for Candy darter (*Etheostoma osburni*) that is listed as near threatened on the IUCN red list [73].

We recommend the use of boosted regression tree models to select key environmental variables by measures of variable importance and evaluation of how a species responds to each environmental gradient by partial dependence curves. Partial dependence curves capture thresholds particularly well (Fig 4), and these are ubiquitous in species habitat relationships. Machine learning techniques developed in the last two decades have some attractive features, such as controlling multicollinearity [39, 44] and being applicable for the case where the number of variables exceeds sample size [28]. However, statistical machine learning techniques tend to over-fit data and produce complicated models with high-dimension interactions, making the model vulnerable in independent validation and prediction, as illustrated with Random Forests [74]. Our results revealed that the BRT model, which have improvements over Random Forest, also tend to over-fit, particularly when the sample size was small. In this study, the BRT and logistic models did not differ significantly in AUC in the validation, although BRT outperformed in the fitting. For the Snake River Basin where the sample size was relatively small, the validation AUC of Lasso logistic model was even higher than the BRT models. Thus, there is a tradeoff to make between potentially over-fitting a model and obtaining more versatile model outputs when sample size is small. The differing behavior of training and validation AUC observed in this study also demonstrates that only reporting model performance in the training or fitting could be misleading, particularly in studies comparing performance of different modeling approaches [1, 75, 76]. While acknowledging that ecologists will have to continue to find ways to work efficiently with presence-only data, we also reinforce growing calls that presence-absence models should be used whenever absence records are available [14, 20, 76]. Even the most powerful presence-only model, MaxEnt, lacks the ability to estimate species prevalence for accurate statistical inference [22], and to adequately evaluate model performance because no true absence are included. Our results show that it would be inefficient not to use the carefully inferred absence data and instead model distributions with a presence-only technique.

It is suggested to explicate spatial autocorrelation and association thereof with environmental predictors in modeling species distribution and assembly patterns [70, 77]. Incorporating spatial autocorrelation improved model accuracy indicated by the ANCOVA in our study (Table 2), particularly for a few fish species in the Brazos River Basin. Including environmental predictors (e.g., temperature, elevation, land use) that spatially auto-correlate may have already removed the spatial dependence in the residuals of the non-spatial model, so adding spatial eigenvectors from the PCNM would not improve the model performance in the New River, Illinois River, and Snake River basins. Theoretically, it is equivalent to the situation that adding covariates highly correlated with the covariates already in the model would not be beneficial. However, our results showed that the suitable fish habitat delineated and predicted distribution of a species may change after spatializing the environmental variables (i.e., a process that detaches spatial information for the environmental variables), although the model performance in terms of AUC would not increase much. The “spatialization” technique utilized in this study essentially filtered the built-in spatial components in each predictor variables, so the variable contribution and rank, and species-habitat relationship are more robust in the spatial models.

Through a bootstrapping resampling procedure on the real data, we confirmed that the effect of prevalence on the model fitting could be confounded by the fact that the variance of the Bernoulli random variable is highest when p = 0.5 and lowest at the extremes. The fitting AUC exhibited a U-shaped response to the prevalence (Fig 3), corroborating observations based on simulated data [78, 79]. The model performance measured by cross-validation AUC was not clearly affected by the prevalence compared to the consistent effect on training AUC, suggesting that cross-validation is essential especially when methods or species are being compared. In addition, we showed that decreasing the total sample size for common species resulted in increased AUC in the model fitting. This sample size effect may be the result of reduced variance in the response when sample size is small and analogous to the over-fitting in linear regression when the number of predictors is close to the sample size. Conclusively, this study provides support for both the ecological (habitat specificity) and statistical (variance of Bernoulli response) basis of rare species tending to have better model performance.

Our results corroborate previous studies that hydrology, climate, land form and cover are key factors that determine distribution of stream fish [16, 80]. It was important initially to include predictors in various habitat categories (e.g., hydrology, stream geomorphology, climate, and anthropogenic impacts) since the biological and ecological traits for most rare non-game species are not well known. Using incomplete set of environmental variables would produce unreliable and misspecified models with the problem of lack of fit, which in turn either overestimate or underestimate species niche and distribution range. Our models demonstrate that none of the broad categories of habitat factors dominantly determined the distribution of these 76 fish species across the United States, and none should be excluded apriori in future species distribution models. Statistical techniques, such as tuning in the Lasso or ridge regression [38], importance ranking and built-in validation in machine-learning models [39, 44], are available to fine-tune the set of predictor variables, so that over-parameterization and multicollinearity should not be a major concern.

## Supporting Information

S1 File The development of the *IchthyMaps* historical metacommunity database. The approach of inferring species absence from historical presences is introduced in this file.
(DOCX)

S2 File The fish presence records in the four selected basins (i.e., New River, Illinois River, Brazos River, and Snake River. These raw data were derived from the *IchthyMaps* database.
(XLSX)

S3 File This file contains Table A-D, and Figure A-B. A table listing the fish species modeled in this study **(Table A)**. Summary of performance (in terms of AUC) of logistic models with Lasso regularization (LM) and boosted regression tree (BRT) models in the training process (_train) and cross validation (_cv) for the 76 fish species in four river basins (BR-Brazos River, IL-Illinois River, NR-New River, SN-Snake River) **(Table B)**. A summary on the key habitat factors for each of the 76 stream fish species in four river basins (i.e., BR-Brazos River, IL-Illinois River, NR-New River, SN-Snake River) in the non-spatial boosted regression tree (BRT) models **(Table C)**. An illustration of inferring absences based on historical fish presence records **(Figure A)**. Relationship of model performance and species prevalence **(Figure B)**.
(DOCX)
