# Supplementary material for: Using Historical Atlas Data to Develop High-Resolution Distribution Models of Freshwater Fishes
Source: PLoS One. 2015 Jun 15;10(6):e0129995. doi: 10.1371/journal.pone.0129995 (PMC4468192; doi:10.1371/journal.pone.0129995)
Supplement: S1 File — The approach of inferring species absence from historical presences is introduced in this file. (DOCX) [file pone.0129995.s001.docx]

**Supplementary Information (S1 File)**

**The development of the *IchthyMaps* historical metacommunity database**

The species presence and absence data used to develop species distribution models in this study were derived from the *IchthyMaps* database. This database, a product of the United States Geological Survey Aquatic Gap Analysis Program, contains 606,550 fish presence records in 224,305 NHDplusV2 (National Hydrography Dataset plus version 2) inter-confluence stream segments. We developed the *IchthyMaps* database by collating and synthesizing species occurrence records from published atlases of freshwater fishes throughout the conterminous United States. We determined the general time frame when the occurrence records were obtained by referring to the books’ prefaces or contacting the authors. Other information (e.g., method of collection, and sampling effort) was usually not specified for individual occurrence records in these atlases. Majority of the fish distribution atlases collected were published during 1970 to 1990 and presence records in those atlases reflected sampling during 1950 to 1990, by seining and electro-fishing methods. Therefore, we considered occurrence records up to 1990 as a practical cutoff for historical distributions, with the assumption that overall habitat and fish community of each stream segment did not change dramatically during this era compared to any changes that might have happened since 1990 or changes (e.g., due to global warming and species invasions) that could be of particular research and conservation interest in the future.

The work flow of deriving fish occurrences from atlases is described in five steps: 1) scanning individual atlases of species occurrences, 2) geo-rectifying and geo-referencing digitized atlases, 3) extracting occurrence records, 4) spatially joining the occurrence records to networks at different scales, and 5) integrating presence records to form the metacommunity database.

*Step 1:* To obtain paper maps, we removed the maps from each book for all the states and regions where atlases were of usable detail and quality, for efficient creation of undistorted, high-resolution maps. For example, in this study, the atlases used to derive species occurrence records included: Atlas of North American Freshwater Fishes [1], Fishes of Montana [2], the Fishes of Illinois [3], Fishes of Idaho [4], Handbook of darters [5], the freshwater fishes of North Carolina [6], Freshwater fishes of Virginia [7], and the Fishes of West Virginia [8]. Each batch (book) of species occurrence atlases was digitized into raster layers through scanning and stored separately. Most printed maps of fish species presence used in this project were displayed on a base stream network map of a resolution that was very close to the NHDPlusV2, i.e., approximately 1:100,000.

*Step 2:* The digitized species occurrence atlases were then geo-rectified and geo-referenced to have the same coordinate and projection system as the NHDPlusV2 base map in ArcInfo (version 10). Geo-referencing many thousands of raster maps could be very time-consuming due to base maps of different sources, maps showing only sections of the country, and varied types of distortions in the scanning process. Therefore, we developed book-by-book templates with optimal coordinate and projection systems that greatly speeded up this process. After geo-referencing the points on the maps to stream segments, the segments became the smallest sampling units for locations where we consider fish sampling to have previously occurred.

*Step 3:* Presence records of each species, usually marked as dots or circles on the maps, were extracted by Feature Analyst, an automated feature extraction extension for ArcInfo (Textron Systems, Providence, RI). Feature Analyst allows rapid and accurate digitizing of target vector features from raster layers (e.g., occurrence points from geo-referenced maps in this case). We began by creating a shapefile of point feature class for each geo-referenced atlas, and providing Feature Analyst with a set of training features (e.g., dots, circles, triangles) matching the symbols for species occurrence, depending on symbols used in the original atlases. After setting up the learning parameters (e.g., searching extent, tolerance of similarity), feature analyst automatically searched symbols similar to those in the training set of the targeted species occurrence map. After running Feature Analyst, a technician inspected and adjusted the output point shapefile by adding any neglected points and removing false-positive errors. Multiple training and learning were iterated when multiple symbols corresponding to different time frames of data collection were used in a single map or when dense occurrence points existed. A point shapefile of occurrences for each species from each book was ultimately stored in a folder of map sources.

*Step 4:* Once shapefiles had been created for all scanned atlases, we used python scripts (Python 2.7) to populate the scientific name of each species and add latitude and longitude coordinates for each point in the attribute table of the species occurrence point shapefile. The updated attribute tables containing species name and latitude and longitude coordinates were integrated by merging all rows of records for each species. It turned out that integrating different GIS layers and all records for a single species through processing attribute tables was much more efficient than merging thousands of shapefiles in ArcGIS. We imported the “.DBF” files (i.e., attribute tables) associated with the presence point shapefiles into the R program iteratively to join the imported tables by row in a ‘for’ loop. As a result, all the presence records were integrated into a table with three columns (species’ scientific name, latitude, and longitude of presence). We named this table as all-presence tables because it synthesized all presence records of fish species we collated from the historical atlases. We spatially joined presence records in this table to the NHDPlusV2 inter-confluence stream segments, HUC (hydrologic unit code) 8-digit and 12-digit watersheds in ArcGIS, so potential users can conveniently query the database at different scales.

*Step 5*: To construct a metacommunity matrix for a region, one needs to first query the presence records of the region from the *Ichthymap* database (for example, Matrix A in Figure A in S3 File). Those sampled segments with at least one presence of a non-game species recorded and no presence record of a focal species were designated as absences for that species. Game fish species, which are mostly sampled in targeted species surveys were excluded from inferring the absence of other species. The created metacommunity matrix is essentially a presence-absence matrix (Matrix B in Figure A in S3 File) with study units (NHD segment, or HUC watershed) as the row names and species as the column names. Each row of the presence-absence matrix represents co-occurrences in a study unit of species belonging to the regional pool, and a column lists the recorded presences and inferred absences of a particular species across all the previously-sampled study units. Creating a presence-absence matrix from the all-presence table can be done with the “xtabs” function of R.

**References in S1 File:**

1. Lee DS, SP Platania, G H Burgess (1980) Atlas of North American Freshwater Fishes. North Carolina State Museum of Natural History, Raleigh, North Carolina. ‎

2. Brown CJD (1971) Fishes of Montana. Montana State University, Bozeman, Montana.

3. Smith PW (1979) The Fishes of Illinois. University of Illinois, Champaign, Illinois.

4. Simpson JC, Wallace RL (1982) Fishes of Idaho. University Press of Idaho, Boise, Mississippi.

5. Page LM (1983) Handbook of darters. Tfh Publications Incorporated, Neptune, New Jersey.‎

6. Menhinick EF (1991) The Freshwater Fishes of North Carolina. North Carolina Wildlife Resources Commission, Raleigh, North Carolina.‎

7. Jenkins RE, Burkhead NM (1994) Freshwater Fishes of Virginia. American Fisheries Society, Bethesda, Maryland.

8. Stauffer JR, Jr., Boltz JM, White LR (1995) The Fishes of West Virginia. Proceedings of the Academy of Natural Sciences of Philadelphia 146: 1-389.
