# Supplementary material for: Using Historical Atlas Data to Develop High-Resolution Distribution Models of Freshwater Fishes
Source: PLoS One. 2015 Jun 15;10(6):e0129995. doi: 10.1371/journal.pone.0129995 (PMC4468192; doi:10.1371/journal.pone.0129995)

**Supplementary information (S3 File)**

**Table A. A list of the fish species modeled in this study.** The observed prevalence of the fish species in the four river basins (NR-New River, BR-Brazos River, IL-Illinois River, SN-Snake River) is provided. The rarity classification of the selected species were obtained from Pritt and Frimpong’s [36] implementation of Rabinowitz [35]. Rarity types are eight combinations of three dimensions (i.e., Dimension 1- range extent, Dimension 2- habitat speciﬁcity, and Dimension 3- local population size) in the rarity classiﬁcation framework [35]. Type (A) is common across three dimensions; Type (B) is rare in the Dimension 3; Type (C) is rare in the Dimension 1; Type (D) is rare in the Dimension 1 and 3; Type (E) is rare in the Dimension 2; Type (F) is rare in the Dimension 2 and 3; Type (G) is rare in the Dimension 1 and 2; and Type (H) is rare across all three dimensions.

| **Scientific name** | **Family** | **Common name** | **Rarity**  **type** | **Basin** | **Prevalence** |
| --- | --- | --- | --- | --- | --- |
| *Acrocheilus alutaceus* | Cyprinidae | Chiselmouth | A | SN | 0.077 |
| *Ameiurus natalis* | Ictaluridae | Yellow bullhead | A | BR | 0.099 |
| *Aphredoderus sayanus* | Aphredoderidae | Pirate perch | A | IL | 0.033 |
| *Campostoma anomalum* | Cyprinidae | Central stoneroller | A | NR | 0.466 |
| *Campostoma anomalum* | Cyprinidae | Central stoneroller | A | BR | 0.222 |
| *Catostomus columbianus* | Catostomidae | Bridgelip sucker | A | SN | 0.185 |
| *Catostomus commersonii* | Catostomidae | White sucker | A | NR | 0.383 |
| *Catostomus commersonii* | Catostomidae | White sucker | A | IL | 0.276 |
| *Cottus bairdii* | Cottidae | Mottled sculpin | A | NR | 0.195 |
| *Cottus bairdii* | Cottidae | Mottled sculpin | A | SN | 0.318 |
| *Cottus carolinae* | Cottidae | Banded sculpin | A | NR | 0.047 |
| *Cottus confusus* | Cottidae | Shorthead sculpin | A | SN | 0.051 |
| *Cyprinella galactura* | Cyprinidae | Whitetail shiner | A | NR | 0.045 |
| *Cyprinella lutrensis* | Cyprinidae | Red shiner | A | IL | 0.256 |
| *Cyprinella venusta* | Cyprinidae | Blacktail shiner | A | BR | 0.102 |
| *Dorosoma petenense* | Clupeidae | Threadfin shad | A | BR | 0.102 |
| *Etheostoma blennioides* | Percidae | Greenside darter | A | NR | 0.27 |
| *Etheostoma caeruleum* | Percidae | Rainbow darter | A | NR | 0.042 |
| *Etheostoma exile* | Percidae | Iowa darter | A | IL | 0.021 |
| *Etheostoma nigrum* | Percidae | Johnny darter | A | IL | 0.287 |
| *Fundulus notatus* | Fundulidae | Blackstripe topminnow | A | IL | 0.115 |
| *Gambusia affinis* | Poeciliidae | Western mosquitofish | A | BR | 0.454 |
| *Hypentelium nigricans* | Catostomidae | Northern hog sucker | A | NR | 0.456 |
| *Ictalurus punctatus* | Ictaluridae | Channel catfish | A | BR | 0.294 |
| *Lepisosteus osseus* | Lepisosteidae | Longnose gar | A | BR | 0.102 |
| *Lepomis humilis* | Centrarchidae | Orangespotted sunfish | A | BR | 0.372 |
| *Lepomis megalotis* | Centrarchidae | Longear sunfish | A | BR | 0.174 |
| *Luxilus chrysocephalus* | Cyprinidae | Striped shiner | A | IL | 0.234 |
| *Luxilus chrysocephalus* | Cyprinidae | Striped shiner | A | NR | 0.056 |
| *Luxilus cornutus* | Cyprinidae | Common shiner | A | IL | 0.068 |
| *Menidia beryllina* | Atherinopsidae | Inland silverside | A | BR | 0.143 |
| *Nocomis biguttatus* | Cyprinidae | Hornyhead chub | A | IL | 0.206 |
| *Nocomis leptocephalus* | Cyprinidae | Bluehead chub | A | NR | 0.188 |
| *Notropis atherinoides* | Cyprinidae | Emerald shiner | A | IL | 0.101 |
| *Notropis dorsalis* | Cyprinidae | Bigmouth shiner | A | IL | 0.279 |
| *Notropis hudsonius* | Cyprinidae | Spottail shiner | A | IL | 0.062 |
| *Notropis rubellus* | Cyprinidae | Rosyface shiner | A | NR | 0.268 |
| *Notropis stramineus* | Cyprinidae | Sand shiner | A | IL | 0.253 |
| *Notropis volucellus* | Cyprinidae | Mimic shiner | A | NR | 0.167 |
| *Noturus gyrinus* | Ictaluridae | Tadpole madtom | A | BR | 0.113 |
| *Pimephales notatus* | Cyprinidae | Bluntnose minnow | A | NR | 0.301 |
| *Pimephales promelas* | Cyprinidae | Fathead minnow | A | BR | 0.106 |
| *Pimephales vigilax* | Cyprinidae | Bullhead minnow | A | BR | 0.628 |
| *Prosopium williamsoni* | Salmonidae | Mountain whitefish | A | SN | 0.097 |
| *Ptychocheilus oregonensis* | Cyprinidae | Northern pikeminnow | A | SN | 0.103 |
| *Rhinichthys cataractae* | Cyprinidae | Longnose dace | A | SN | 0.179 |
| *Rhinichthys cataractae* | Cyprinidae | Longnose dace | A | NR | 0.242 |
| *Richardsonius balteatus* | Cyprinidae | Redside shiner | A | SN | 0.231 |
| *Amia calva* | Amiidae | Bowfin | B | IL | 0.032 |
| *Etheostoma microperca* | Percidae | Least darter | B | IL | 0.036 |
| *Lythrurus ardens* | Cyprinidae | Rosefin shiner | B | NR | 0.083 |
| *Notropis buccatus* | Cyprinidae | Silverjaw minnow | B | IL | 0.044 |
| *Notropis buccatus* | Cyprinidae | Silverjaw minnow | B | NR | 0.054 |
| *Opsopoeodus emiliae* | Cyprinidae | Pugnose minnow | B | IL | 0.029 |
| *Opsopoeodus emiliae* | Cyprinidae | Pugnose minnow | B | BR | 0.126 |
| *Campostoma oligolepis* | Cyprinidae | Largescale stoneroller | C | IL | 0.021 |
| *Carpiodes velifer* | Catostomidae | Highfin carpsucker | C | IL | 0.051 |
| *Cottus beldingii* | Cottidae | Paiute sculpin | C | SN | 0.087 |
| *Cottus rhotheus* | Cottidae | Torrent sculpin | C | SN | 0.041 |
| *Cyprinella spiloptera* | Cyprinidae | Spotfin shiner | C | IL | 0.091 |
| *Cyprinella spiloptera* | Cyprinidae | Spotfin shiner | C | NR | 0.155 |
| *Etheostoma chlorosoma* | Percidae | Bluntnose darter | C | IL | 0.046 |
| *Etheostoma chlorosoma* | Percidae | Bluntnose darter | C | BR | 0.092 |
| *Etheostoma spectabile* | Percidae | Orangethroat darter | C | IL | 0.134 |
| *Etheostoma spectabile* | Percidae | Orangethroat darter | C | BR | 0.113 |
| *Hybognathus nuchalis* | Cyprinidae | Mississippi silvery minnow | C | IL | 0.067 |
| *Ictiobus bubalus* | Catostomidae | Smallmouth buffalo | C | IL | 0.044 |
| *Notropis buchanani* | Cyprinidae | Ghost shiner | C | BR | 0.119 |
| *Percina phoxocephala* | Percidae | Slenderhead darter | C | IL | 0.097 |
| *Chrosomus erythrogaster* | Cyprinidae | Southern redbelly dace | C | IL | 0.062 |
| *Etheostoma asprigene* | Percidae | Mud darter | D | IL | 0.052 |
| *Etheostoma gracile* | Percidae | Slough darter | D | BR | 0.177 |
| *Percina sciera* | Percidae | Dusky darter | D | BR | 0.160 |
| *Catostomus ardens* | Catostomidae | Utah sucker | E | SN | 0.062 |
| *Luxilus cerasinus* | Cyprinidae | Crescent shiner | E | NR | 0.059 |
| *Nocomis platyrhynchus* | Cyprinidae | Bigmouth chub | E | NR | 0.289 |
| *Notropis rubricroceus* | Cyprinidae | Saffron shiner | E | NR | 0.033 |
| *Percina oxyrhynchus* | Percidae | Sharpnose darter | E | NR | 0.111 |
| *Percina roanoka* | Percidae | Roanoke darter | E | NR | 0.064 |
| *Chrosomus oreas* | Cyprinidae | Mountain redbelly dace | E | NR | 0.191 |
| *Etheostoma kanawhae* | Percidae | Kanawha darter | F | NR | 0.099 |
| *Etheostoma osburni* | Percidae | Candy darter | F | NR | 0.111 |
| *Notropis scabriceps* | Cyprinidae | New River shiner | F | NR | 0.174 |
| *Luxilus albeolus* | Cyprinidae | White shiner | G | NR | 0.151 |
| *Exoglossum laurae* | Cyprinidae | Tonguetied minnow | H | NR | 0.191 |
| *Phenacobius teretulus* | Cyprinidae | Kanawha minnow | H | NR | 0.113 |
| *Chrosomus tennesseensis* | Cyprinidae | Tennessee dace | H | NR | 0.129 |

**Table B. Summary of performance (in terms of AUC) of logistic regression models with Lasso regularization (LM) and boosted regression tree (BRT) models in the training process (_train) and cross validation (_cv) for the 76 fish species in the four selected river basins (BR-Brazos River, IL-Illinois River, NR-New River, SN-Snake River).** “BRT_s_cv” and “LM_s_cv” mean that spatial autocorrelation was incorporated in the models. The common name and family of each species are given in Table A.

| **Species** | **Basin** | | **Model results** | | | | | | | | | | | |
| --- | --- | --- | --- | --- | --- | --- | --- | --- | --- | --- | --- | --- | --- | --- |
|  |  |  | **BRT_train** | | **BRT_cv** | | **BRT_s_cv** | | **LM_train** | | **LM_cv** | | **LM_s_cv** | |
| *Acrocheilus alutaceus* | SN | 0.945 | | 0.692 | | 0.728 | | 0.889 | | 0.816 | | 0.847 | |  |
| *Ameiurus natalis* | BR | 0.968 | | 0.658 | | 0.909 | | 0.765 | | 0.710 | | 0.910 | |  |
| *Aphredoderus sayanus* | IL | 0.972 | | 0.672 | | 0.737 | | 0.773 | | 0.730 | | 0.655 | |  |
| *Campostoma anomalum* | NR | 0.998 | | 0.721 | | 0.644 | | 0.689 | | 0.710 | | 0.681 | |  |
| *Campostoma anomalum* | BR | 0.981 | | 0.894 | | 0.728 | | 0.883 | | 0.836 | | 0.741 | |  |
| *Catostomus columbianus* | SN | 0.967 | | 0.705 | | 0.662 | | 0.823 | | 0.737 | | 0.706 | |  |
| *Catostomus commersonii* | NR | 0.746 | | 0.587 | | 0.565 | | 0.653 | | 0.619 | | 0.573 | |  |
| *Catostomus commersonii* | IL | 0.765 | | 0.600 | | 0.594 | | 0.598 | | 0.559 | | 0.582 | |  |
| *Cottus bairdii* | NR | 0.866 | | 0.621 | | 0.654 | | 0.678 | | 0.615 | | 0.655 | |  |
| *Cottus bairdii* | SN | 0.938 | | 0.679 | | 0.735 | | 0.803 | | 0.719 | | 0.741 | |  |
| *Cottus carolinae* | NR | 0.999 | | 0.969 | | 0.934 | | 0.500 | | 0.967 | | 0.944 | |  |
| *Cottus confusus* | SN | 0.992 | | 0.648 | | 0.883 | | 0.840 | | 0.703 | | 0.748 | |  |
| *Cyprinella galactura* | NR | 0.998 | | 0.882 | | 0.882 | | 0.933 | | 0.897 | | 0.917 | |  |
| *Cyprinella lutrensis* | IL | 0.838 | | 0.705 | | 0.696 | | 0.731 | | 0.715 | | 0.716 | |  |
| *Cyprinella venusta* | BR | 0.939 | | 0.834 | | 0.928 | | 0.826 | | 0.804 | | 0.935 | |  |
| *Dorosoma petenense* | BR | 0.964 | | 0.852 | | 0.913 | | 0.932 | | 0.845 | | 0.927 | |  |
| *Etheostoma blennioides* | NR | 0.890 | | 0.792 | | 0.717 | | 0.725 | | 0.730 | | 0.717 | |  |
| *Etheostoma caeruleum* | NR | 0.997 | | 0.939 | | 0.958 | | 0.500 | | 0.973 | | 0.957 | |  |
| *Etheostoma exile* | IL | 0.996 | | 0.896 | | 0.857 | | 0.500 | | 0.909 | | 0.842 | |  |
| *Etheostoma nigrum* | IL | 0.798 | | 0.621 | | 0.576 | | 0.644 | | 0.639 | | 0.610 | |  |
| *Fundulus notatus* | IL | 0.825 | | 0.639 | | 0.646 | | 0.712 | | 0.674 | | 0.684 | |  |
| *Gambusia affinis* | BR | 0.912 | | 0.650 | | 0.623 | | 0.701 | | 0.572 | | 0.593 | |  |
| *Hypentelium nigricans* | NR | 0.737 | | 0.561 | | 0.546 | | 0.623 | | 0.548 | | 0.569 | |  |
| *Ictalurus punctatus* | BR | 0.925 | | 0.745 | | 0.679 | | 0.711 | | 0.721 | | 0.702 | |  |
| *Lepisosteus osseus* | BR | 0.985 | | 0.792 | | 0.939 | | 0.910 | | 0.775 | | 0.931 | |  |
| *Lepomis humilis* | BR | 0.870 | | 0.659 | | 0.838 | | 0.625 | | 0.634 | | 0.831 | |  |
| *Lepomis megalotis* | BR | 0.889 | | 0.622 | | 0.724 | | 0.618 | | 0.572 | | 0.760 | |  |
| *Luxilus chrysocephalus* | IL | 0.801 | | 0.697 | | 0.667 | | 0.690 | | 0.684 | | 0.669 | |  |
| *Luxilus chrysocephalus* | NR | 0.998 | | 0.954 | | 0.935 | | 0.500 | | 0.953 | | 0.940 | |  |
| *Luxilus cornutus* | IL | 0.974 | | 0.905 | | 0.895 | | 0.918 | | 0.908 | | 0.897 | |  |
| *Menidia beryllina* | BR | 0.923 | | 0.701 | | 0.676 | | 0.708 | | 0.746 | | 0.714 | |  |
| *Nocomis biguttatus* | IL | 0.782 | | 0.639 | | 0.615 | | 0.649 | | 0.651 | | 0.649 | |  |
| *Nocomis leptocephalus* | NR | 0.862 | | 0.632 | | 0.711 | | 0.639 | | 0.585 | | 0.725 | |  |
| *Notropis atherinoides* | IL | 0.918 | | 0.784 | | 0.764 | | 0.812 | | 0.775 | | 0.762 | |  |
| *Notropis dorsalis* | IL | 0.863 | | 0.642 | | 0.604 | | 0.706 | | 0.664 | | 0.651 | |  |
| *Notropis hudsonius* | IL | 0.972 | | 0.821 | | 0.814 | | 0.500 | | 0.826 | | 0.831 | |  |
| *Notropis rubellus* | NR | 0.822 | | 0.648 | | 0.621 | | 0.692 | | 0.624 | | 0.650 | |  |
| *Notropis stramineus* | IL | 0.752 | | 0.584 | | 0.583 | | 0.620 | | 0.571 | | 0.582 | |  |
| *Notropis volucellus* | NR | 0.868 | | 0.725 | | 0.719 | | 0.771 | | 0.773 | | 0.722 | |  |
| *Noturus gyrinus* | BR | 0.950 | | 0.781 | | 0.847 | | 0.835 | | 0.827 | | 0.887 | |  |
| *Pimephales notatus* | NR | 0.934 | | 0.745 | | 0.773 | | 0.725 | | 0.719 | | 0.788 | |  |
| *Pimephales promelas* | BR | 0.948 | | 0.668 | | 0.675 | | 0.757 | | 0.740 | | 0.786 | |  |
| *Pimephales vigilax* | BR | 0.903 | | 0.685 | | 0.617 | | 0.670 | | 0.647 | | 0.580 | |  |
| *Prosopium williamsoni* | SN | 0.903 | | 0.591 | | 0.563 | | 0.733 | | 0.783 | | 0.578 | |  |
| *Ptychocheilus oregonensis* | SN | 0.985 | | 0.839 | | 0.822 | | 0.894 | | 0.909 | | 0.914 | |  |
| *Rhinichthys cataractae* | SN | 0.887 | | 0.599 | | 0.525 | | 0.836 | | 0.661 | | 0.538 | |  |
| *Rhinichthys cataractae* | NR | 0.754 | | 0.578 | | 0.574 | | 0.632 | | 0.604 | | 0.596 | |  |
| *Richardsonius balteatus* | SN | 0.843 | | 0.569 | | 0.600 | | 0.720 | | 0.627 | | 0.634 | |  |
| *Amia calva* | IL | 0.977 | | 0.831 | | 0.806 | | 0.500 | | 0.778 | | 0.824 | |  |
| *Etheostoma microperca* | IL | 0.982 | | 0.880 | | 0.898 | | 0.500 | | 0.868 | | 0.883 | |  |
| *Lythrurus ardens* | NR | 0.919 | | 0.758 | | 0.774 | | 0.842 | | 0.714 | | 0.785 | |  |
| *Notropis buccatus* | IL | 0.983 | | 0.848 | | 0.887 | | 0.500 | | 0.799 | | 0.888 | |  |
| *Notropis buccatus* | NR | 1.000 | | 0.967 | | 0.903 | | 0.500 | | 0.971 | | 0.951 | |  |
| *Opsopoeodus emiliae* | IL | 0.976 | | 0.790 | | 0.715 | | 0.850 | | 0.838 | | 0.746 | |  |
| *Opsopoeodus emiliae* | BR | 0.972 | | 0.880 | | 0.749 | | 0.942 | | 0.909 | | 0.828 | |  |
| *Campostoma oligolepis* | IL | 0.998 | | 0.818 | | 0.833 | | 0.862 | | 0.728 | | 0.789 | |  |
| *Carpiodes velifer* | IL | 0.946 | | 0.727 | | 0.712 | | 0.749 | | 0.714 | | 0.694 | |  |
| *Cottus beldingii* | SN | 0.993 | | 0.719 | | 0.643 | | 0.769 | | 0.815 | | 0.793 | |  |
| *Cottus rhotheus* | SN | 0.998 | | 0.664 | | 0.750 | | 0.956 | | 0.817 | | 0.810 | |  |
| *Cyprinella spiloptera* | IL | 0.935 | | 0.717 | | 0.761 | | 0.807 | | 0.753 | | 0.734 | |  |
| *Cyprinella spiloptera* | NR | 0.926 | | 0.793 | | 0.817 | | 0.803 | | 0.789 | | 0.809 | |  |
| *Etheostoma chlorosoma* | IL | 0.958 | | 0.830 | | 0.788 | | 0.826 | | 0.804 | | 0.825 | |  |
| *Etheostoma chlorosoma* | BR | 0.998 | | 0.946 | | 0.868 | | 0.966 | | 0.936 | | 0.860 | |  |
| *Etheostoma spectabile* | IL | 0.898 | | 0.677 | | 0.636 | | 0.705 | | 0.648 | | 0.652 | |  |
| *Etheostoma spectabile* | BR | 0.982 | | 0.892 | | 0.858 | | 0.909 | | 0.892 | | 0.876 | |  |
| *Hybognathus nuchalis* | IL | 0.889 | | 0.750 | | 0.742 | | 0.762 | | 0.759 | | 0.750 | |  |
| *Ictiobus bubalus* | IL | 0.956 | | 0.837 | | 0.842 | | 0.887 | | 0.867 | | 0.819 | |  |
| *Notropis buchanani* | BR | 0.929 | | 0.765 | | 0.868 | | 0.791 | | 0.774 | | 0.899 | |  |
| *Percina phoxocephala* | IL | 0.928 | | 0.743 | | 0.677 | | 0.801 | | 0.747 | | 0.640 | |  |
| *Chrosomus erythrogaster* | IL | 0.945 | | 0.744 | | 0.697 | | 0.813 | | 0.737 | | 0.732 | |  |
| *Etheostoma asprigene* | IL | 0.970 | | 0.823 | | 0.785 | | 0.854 | | 0.838 | | 0.825 | |  |
| *Etheostoma gracile* | BR | 0.981 | | 0.892 | | 0.724 | | 0.928 | | 0.916 | | 0.692 | |  |
| *Percina sciera* | BR | 0.940 | | 0.782 | | 0.861 | | 0.763 | | 0.736 | | 0.832 | |  |
| *Catostomus ardens* | SN | 0.999 | | 0.846 | | 0.908 | | 0.922 | | 0.921 | | 0.954 | |  |
| *Luxilus cerasinus* | NR | 0.987 | | 0.751 | | 0.771 | | 0.876 | | 0.728 | | 0.785 | |  |
| *Nocomis platyrhynchus* | NR | 0.817 | | 0.668 | | 0.600 | | 0.700 | | 0.625 | | 0.604 | |  |
| *Notropis rubricroceus* | NR | 0.973 | | 0.798 | | 0.772 | | 0.895 | | 0.771 | | 0.893 | |  |
| *Percina oxyrhynchus* | NR | 0.888 | | 0.718 | | 0.752 | | 0.789 | | 0.760 | | 0.749 | |  |
| *Percina roanoka* | NR | 0.991 | | 0.844 | | 0.831 | | 0.730 | | 0.838 | | 0.791 | |  |
| *Chrosomus oreas* | NR | 0.895 | | 0.678 | | 0.776 | | 0.655 | | 0.659 | | 0.725 | |  |
| *Etheostoma kanawhae* | NR | 0.945 | | 0.747 | | 0.812 | | 0.770 | | 0.744 | | 0.785 | |  |
| *Etheostoma osburni* | NR | 0.980 | | 0.869 | | 0.835 | | 0.908 | | 0.887 | | 0.858 | |  |
| *Notropis scabriceps* | NR | 0.877 | | 0.695 | | 0.666 | | 0.731 | | 0.647 | | 0.647 | |  |
| *Luxilus albeolus* | NR | 0.857 | | 0.667 | | 0.727 | | 0.707 | | 0.702 | | 0.745 | |  |
| *Exoglossum laurae* | NR | 0.913 | | 0.665 | | 0.704 | | 0.771 | | 0.698 | | 0.672 | |  |
| *Phenacobius teretulus* | NR | 0.894 | | 0.750 | | 0.774 | | 0.764 | | 0.759 | | 0.782 | |  |

**Table C. A summary of the key habitat factors predicting the distribution of each of the 76 stream fish species in four river basins (i.e., BR-Brazos River, IL-Illinois River, NR-New River, SN-Snake River) in the non-spatial boosted regression tree (BRT) models.** The number in the bracket is the measure of variable importance or percentage of contribution by the BRT models [46]. The response of the each species to important habitat factors were evaluated using partial dependence plots [46]. We use “⤴” to represent a clear positive relationship, and “⤵” for a negative relationship, and “~” for a complicated non-linear relationships (e.g., polynomial, hinge). For instance, “TM (39) ⤴” means that annual mean temperature (TM) was the most important and positive environmental variable that contributed 39.3% in the BRT model for Chiselmouth (*Acrocheilus alutaceus*) in the Snake River basin. The descriptions of environmental predictors are listed in Table 1.

| **Species** | **Basin** | **Key predictors** | | | | |
| --- | --- | --- | --- | --- | --- | --- |
|  |  | **V1** | **V2** | **V3** | **V4** | **V5** |
| *Acrocheilus alutaceus* | SN | TM (39) ⤴ | TMI (9) ~ | PPT (8) ~ | C_WT (7) ~ | BFI (7) ⤵ |
| *Ameiurus natalis* | BR | PPT (10) ⤴ | FHS (9) ~ | BFI (8) ⤴ | SINU (7) ⤵ | DRA (7) ~ |
| *Aphredoderus sayanus* | IL | TMI (16) ⤴ | C_FR (8) ~ | SLP (8) ~ | SINU (7) ~ | PPT (6) ⤴ |
| *Campostoma anomalum* | NR | BFI (68) ~ | TMI (7) ⤵ | C_FR (4) ⤵ | SLP (3) ⤴ | ELE (2) ⤵ |
| *Campostoma anomalum* | BR | BFI (19) ⤴ | PPT (17) ~ | ELE (13) ~ | SLP (9) ⤴ | DRA (7) ~ |
| *Catostomus columbianus* | SN | BFI (21) ⤵ | SLP (12) ⤵ | C_AG (8) ~ | SO (6) ⤵ | MFU (6) ~ |
| *Catostomus commersonii* | NR | BFI (40) ~ | NT (11) ~ | FHS (8) ⤵ | PT (8) ~ | TMI (4) ~ |
| *Catostomus commersonii* | IL | TMI (14) ~ | TM (9) ⤴ | MVU (7) ~ | DRA (6) ~ | SINU (6) ⤴ |
| *Cottus bairdii* | NR | SLP (9) ⤵ | C_WT (9) ~ | MVU (8) ~ | NT (8) ~ | DRA (6) ~ |
| *Cottus bairdii* | SN | BFI (21) ⤴ | PPT (16) ⤴ | D_FR (10) ⤴ | FHS (7) ~ | TMI (6) ⤵ |
| *Cottus carolinae* | NR | TMI (39) ⤵ | TM (20) ⤵ | PPT (14) ⤵ | ELE (5) ⤵ | TMA (4) ⤴ |
| *Cottus confusus* | SN | TMA (24) ~ | TMI (17) ⤵ | D_FR (11) ~ | C_FR (7) ⤴ | FHS (5) ~ |
| *Cyprinella galactura* | NR | TMI (15) ⤴ | PPT (12) ~ | DRA (11) ⤴ | SINU (10) ⤴ | BFI (7) ⤵ |
| *Cyprinella lutrensis* | IL | TMA (35) ⤴ | TM (20) ⤴ | POP (5) ~ | BFI (4) ⤵ | SO (3) ⤴ |
| *Cyprinella venusta* | BR | BFI (35) ⤴ | PPT (18) ~ | SINU (5) ⤵ | MVU (4) ⤴ | DRA (4) ~ |
| *Dorosoma petenense* | BR | DRA (27) ⤴ | MFU (23) ⤴ | BFI (9) ⤵ | C_WT (9) ⤴ | C_AG (3) ~ |
| *Etheostoma blennioides* | NR | DRA (31) ~ | BFI (12) ⤵ | MFU (12) ~ | TMI (10) ⤵ | D_FR (4) ⤴ |
| *Etheostoma caeruleum* | NR | SLP (33) ⤴ | ELE (21) ⤵ | BFI (10) ⤵ | PPT (5) ⤵ | FHS (5) ⤵ |
| *Etheostoma exile* | IL | BFI (20) ⤴ | C_WT (17) ~ | TMI (9) ⤵ | C_AG (8) ⤵ | D_AG (6) ⤵ |
| *Etheostoma nigrum* | IL | ELE (21) ⤴ | NT (11) ~ | PT (10) ~ | BFI (7) ⤴ | POP (5) ~ |
| *Fundulus notatus* | IL | SLP (18) ~ | PT (15) ~ | NT (13) ~ | TMI (7) ⤴ | ELE (6) ⤵ |
| *Gambusia affinis* | BR | C_FR (15) ~ | SLP (10) ⤴ | C_AG (9) ~ | MVU (9) ⤴ | NT (6) ~ |
| *Hypentelium nigricans* | NR | BFI (44) ⤵ | PT (8) ~ | POP (7) ~ | DRA (5) ~ | C_FR (4) ⤴ |
| *Ictalurus punctatus* | BR | MFU (19) ⤴ | DRA (13) ⤴ | MVU (8) ⤴ | PT (6) ⤴ | ELE (6) ~ |
| *Lepisosteus osseus* | BR | D_AG (13) ~ | MFU (11) ~ | FHS (10) ⤵ | POP (9) ~ | DRA (8) ⤵ |
| *Lepomis humilis* | BR | ELE (33) ~ | BFI (11) ⤵ | C_WT (7) ~ | D_FR (6) ⤴ | MFU (4) ⤴ |
| *Lepomis megalotis* | BR | POP (19) ~ | MVU (10) ~ | D_FR (8) ⤴ | TM (7) ⤴ | SLP (7) ~ |
| *Luxilus chrysocephalus* | IL | D_FR (23) ~ | BFI (19) ~ | FHS (12) ⤵ | TMA (9) ~ | TM (4) ⤴ |
| *Luxilus chrysocephalus* | NR | TMI (52) ⤵ | TMA (9) ⤴ | DRA (6) ⤴ | MFU (6) ⤴ | C_UB (5) ~ |
| *Luxilus cornutus* | IL | TM (41) ⤵ | TMI (17) ⤵ | POP (6) ⤴ | PPT (4) ⤵ | D_AG (3) ⤴ |
| *Menidia beryllina* | BR | DRA (18) ⤴ | RDX (16) ~ | C_WT (11) ⤴ | POP (9) ~ | MVU (9) ⤴ |
| *Nocomis biguttatus* | IL | D_AG (23) ~ | SLP (9) ~ | PT (9) ~ | BFI (8) ~ | NT (6) ~ |
| *Nocomis leptocephalus* | NR | BFI (15) ⤴ | SLP (12) ~ | PPT (12) ⤵ | ELE (7) ⤵ | TM (6) ⤴ |
| *Notropis atherinoides* | IL | ELE (36) ⤵ | TMA (10) ⤵ | D_AG (8) ⤵ | C_AG (4) ⤵ | POP (4) ⤴ |
| *Notropis dorsalis* | IL | D_AG (11) ⤴ | TM (9) ⤴ | PPT (7) ⤵ | MFU (7) ~ | TMA (6) ⤴ |
| *Notropis hudsonius* | IL | ELE (22) ⤵ | DRA (9) ⤴ | C_WT (8) ~ | TMI (6) ⤵ | POP (5) ~ |
| *Notropis rubellus* | NR | BFI (19) ⤵ | MFU (15) ~ | TMI (12) ⤵ | TM (12) ~ | PPT (8) ⤴ |
| *Notropis stramineus* | IL | MFU (13) ~ | PT (11) ~ | POP (10) ~ | NT (9) ~ | ELE (8) ⤵ |
| *Notropis volucellus* | NR | MFU (18) ⤴ | DRA (15) ⤴ | BFI (14) ⤵ | MVU (8) ⤴ | TMI (7) ⤵ |
| *Noturus gyrinus* | BR | ELE (24) ⤵ | MVU (7) ⤴ | TM (7) ⤴ | TMI (6) ⤴ | D_AG (5) ⤴ |
| *Pimephales notatus* | NR | BFI (34) ~ | MFU (14) ~ | SLP (7) ⤴ | FHS (5) ⤵ | SINU (5) ⤴ |
| *Pimephales promelas* | BR | RDX (13) ⤴ | POP (11) ~ | C_UB (8) ⤴ | TMI (8) ⤵ | PPT (8) ⤴ |
| *Pimephales vigilax* | BR | MFU (14) ~ | TMA (13) ⤴ | DRA (11) ~ | BFI (8) ⤵ | ELE (6) ⤵ |
| *Prosopium williamsoni* | SN | C_UB (48) ~ | RDX (12) ⤵ | TMA (7) ⤵ | C_AG (5) ⤵ | BFI (3) ⤴ |
| *Ptychocheilus oregonensis* | SN | BFI (53) ~ | ELE (9) ⤵ | SLP (6) ⤵ | C_AG (5) ⤴ | POP (3) ~ |
| *Rhinichthys cataractae* | SN | SINU (42) ⤴ | C_AG (9) ⤵ | BFI (9) ⤴ | DRA (7) ~ | RDX (4) ⤴ |
| *Rhinichthys cataractae* | NR | BFI (46) ~ | ELE (7) ⤵ | C_AG (6) ⤴ | RDX (6) ~ | MVU (5) ⤴ |
| *Richardsonius balteatus* | SN | C_FR (16) ~ | SLP (16) ⤵ | TMA (10) ~ | NT (8) ~ | D_FR (8) ⤵ |
| *Amia calva* | IL | ELE (17) ~ | TM (17) ⤵ | C_UB (10) ~ | C_WT (9) ~ | BFI (6) ~ |
| *Etheostoma microperca* | IL | BFI (42) ⤴ | PPT (8) ⤴ | TMA (8) ⤵ | NT (7) ~ | PT (5) ~ |
| *Lythrurus ardens* | NR | PPT (17) ⤵ | ELE (12) ⤵ | PT (12) ~ | BFI (11) ⤵ | NT (7) ~ |
| *Notropis buccatus* | IL | ELE (17) ⤵ | TMI (15) ⤴ | NT (12) ~ | TM (11) ⤵ | TMA (8) ⤵ |
| *Notropis buccatus* | NR | BFI (50) ~ | SLP (13) ⤴ | C_UB (6) ~ | TM (5) ⤴ | DRA (3) ⤴ |
| *Opsopoeodus emiliae* | IL | MFU (18) ⤴ | ELE (12) ⤵ | C_WT (10) ~ | TMA (6) ~ | TM (5) ~ |
| *Opsopoeodus emiliae* | BR | ELE (43) ~ | POP (7) ~ | PPT (7) ⤴ | SLP (5) ⤴ | FHS (5) ⤵ |
| *Campostoma oligolepis* | IL | ELE (19) ~ | TMI (11) ⤵ | NT (10) ~ | BFI (8) ⤴ | D_AG (8) ⤴ |
| *Carpiodes velifer* | IL | D_FR (16) ~ | TMA (14) ⤴ | MVU (11) ⤴ | C_UB (7) ⤴ | TMI (5) ⤴ |
| *Cottus beldingii* | SN | PPT (30) ⤴ | SLP (10) ⤴ | D_FR (7) ⤵ | BFI (7) ~ | MVU (6) ~ |
| *Cottus rhotheus* | SN | SLP (46) ⤴ | C_FR (22) ⤵ | TMA (6) ⤵ | NT (3) ~ | FHS (3) ~ |
| *Cyprinella spiloptera* | IL | TMA (13) ⤵ | BFI (10) ⤴ | ELE (8) ⤵ | POP (8) ~ | MVU (7) ⤴ |
| *Cyprinella spiloptera* | NR | MFU (18) ~ | BFI (14) ⤵ | DRA (13) ⤴ | PPT (10) ⤵ | ELE (6) ⤵ |
| *Etheostoma chlorosoma* | IL | ELE (42) ⤵ | D_AG (9) ⤵ | TMI (9) ⤴ | NT (7) ~ | BFI (4) ~ |
| *Etheostoma chlorosoma* | BR | PPT (46) ⤴ | TMI (13) ⤴ | SLP (5) ⤴ | C_AG (5) ⤵ | C_FR (4) ⤵ |
| *Etheostoma spectabile* | IL | BFI (13) ~ | PPT (10) ⤴ | TMI (10) ⤵ | ELE (8) ⤴ | SLP (7) ⤴ |
| *Etheostoma spectabile* | BR | BFI (43) ⤴ | TMA (17) ⤴ | SLP (8) ⤴ | C_FR (6) ~ | SINU (4) ⤵ |
| *Hybognathus nuchalis* | IL | ELE (19) ⤵ | D_FR (13) ⤴ | TMA (11) ⤴ | SINU (9) ~ | TM (8) ⤴ |
| *Ictiobus bubalus* | IL | ELE (50) ⤵ | C_AG (9) ⤵ | DRA (6) ~ | D_AG (4) ⤵ | FHS (3) ⤴ |
| *Notropis buchanani* | BR | MFU (37) ⤴ | POP (7) ~ | MVU (6) ~ | TM (4) ~ | DRA (4) ⤴ |
| *Percina phoxocephala* | IL | MFU (12) ~ | PT (12) ~ | SINU (9) ⤴ | TMA (8) ⤴ | DRA (7) ~ |
| *Chrosomus erythrogaster* | IL | TM (20) ⤵ | TMI (11) ⤵ | BFI (8) ⤵ | C_FR (6) ⤴ | TMA (6) ⤴ |
| *Etheostoma asprigene* | IL | ELE (35) ⤵ | POP (10) ~ | TMA (7) ⤴ | C_FR (5) ~ | D_AG (4) ⤵ |
| *Etheostoma gracile* | BR | PPT (32) ⤴ | TMI (15) ⤴ | C_AG (8) ⤵ | ELE (4) ~ | DRA (4) ⤴ |
| *Percina sciera* | BR | MFU (23) ~ | PPT (12) ⤴ | C_AG (7) ⤴ | SLP (7) ⤴ | ELE (6) ⤵ |
| *Catostomus ardens* | SN | BFI (31) ~ | ELE (14) ~ | C_AG (10) ⤴ | POP (8) ~ | D_AG (5) ⤴ |
| *Luxilus cerasinus* | NR | ELE (12) ~ | TMA (11) ⤴ | SINU (10) ⤴ | PPT (7) ~ | MFU (7) ~ |
| *Nocomis platyrhynchus* | NR | BFI (32) ~ | DRA (21) ~ | NT (5) ~ | MFU (4) ~ | MVU (3) ⤵ |
| *Notropis rubricroceus* | NR | C_FR (27) ~ | POP (17) ~ | BFI (15) ⤴ | PPT (8) ⤵ | NT (4) ~ |
| *Percina oxyrhynchus* | NR | MVU (21) ⤴ | MFU (20) ⤴ | DRA (13) ⤴ | POP (7) ~ | ELE (7) ⤵ |
| *Percina roanoka* | NR | DRA (18) ⤴ | MFU (12) ⤴ | TMA (11) ⤵ | BFI (7) ⤴ | POP (7) ~ |
| *Chrosomus oreas* | NR | D_AG (14) ⤴ | SINU (10) ⤴ | PPT (9) ⤵ | TM (8) ⤴ | BFI (8) ⤴ |
| *Etheostoma kanawhae* | NR | SINU (18) ⤴ | BFI (17) ⤴ | POP (10) ~ | C_AG (9) ~ | PPT (9) ⤴ |
| *Etheostoma osburni* | NR | BFI (27) ⤵ | TMI (19) ⤵ | D_FR (12) ~ | PPT (6) ~ | C_UB (4) ⤴ |
| *Notropis scabriceps* | NR | TMI (25) ⤵ | DRA (13) ~ | BFI (13) ⤴ | PPT (7) ⤴ | PT (5) ~ |
| *Luxilus albeolus* | NR | ELE (23) ⤵ | BFI (13) ⤵ | TMA (8) ⤴ | PPT (6) ⤵ | SINU (6) ⤴ |
| *Exoglossum laurae* | NR | MVU (13) ⤵ | POP (8) ~ | TMI (8) ⤵ | SINU (7) ~ | PPT (6) ⤵ |
| *Phenacobius teretulus* | NR | BFI (37) ⤴ | DRA (17) ~ | C_FR (8) ⤵ | MVU (4) ~ | NT (4) ~ |

**Table D. A summary of the key environmental factors predicting the distribution of each of the 76 stream fish species in four river basins (i.e., BR-Brazos River, IL-Illinois River, NR-New River, SN-Snake River) in the spatial boosted regression tree (BRT) models.** In the spatial models, principal coordinate analysis of neighbor matrices, PCNM [32], was used to spatialize environmental variables. The number in the bracket is the measure of variable importance or percentage of contribution to the BRT models [46]. The response of each species to important environmental factors were evaluated using partial dependence plots [46]. We use “⤴” to represent a clear positive relationship, and “⤵” for a negative relationship, and “~” for a complicated non-linear relationships (e.g., polynomial, hinge). The descriptions of environmental predictors are listed in Table 1.

| **Species** | **Basin** | **Key predictors** | | | | |
| --- | --- | --- | --- | --- | --- | --- |
|  |  | **V1** | **V2** | **V3** | **V4** | **V5** |
| *Acrocheilus alutaceus* | SN | TM (19) ⤴ | TMI (18) ⤴ | ELE (11) ⤵ | D_UB (11) ⤵ | POP (9) ⤵ |
| *Ameiurus natalis* | BR | C_FR (22) ⤵ | ELE (21) ⤵ | PPT (7) ⤴ | BFI (7) ⤴ | DRA (7) ~ |
| *Aphredoderus sayanus* | IL | TMI (34) ⤴ | PPT (11) ⤴ | MVU (9) ⤵ | C_WA (4) ~ | PT (4) ⤴ |
| *Campostoma anomalum* | NR | BFI (60) ⤵ | PPT (5) ⤵ | D_AG (4) ~ | POP (3) ⤴ | TMI (3) ⤵ |
| *Campostoma anomalum* | BR | BFI (31) ⤴ | SLP (10) ⤴ | PPT (9) ~ | SO (8) ⤵ | RDX (5) ⤴ |
| *Catostomus columbianus* | SN | C_GR (27) ⤴ | SO (11) ⤵ | DRA (10) ⤵ | TMI (9) ⤴ | BFI (8) ⤵ |
| *Catostomus commersonii* | NR | FHS (24) ⤵ | BFI (23) ⤵ | MVU (10) ⤵ | TMA (8) ~ | ELE (4) ⤵ |
| *Catostomus commersonii* | IL | SINU (12) ⤴ | FHS (9) ~ | C_FR (7) ⤵ | ELE (7) ~ | D_FR (6) ⤵ |
| *Cottus bairdii* | NR | C_WA (15) ⤵ | BFI (10) ⤴ | PT (10) ⤴ | TMI (7) ⤵ | C_FR (6) ~ |
| *Cottus bairdii* | SN | C_GR (17) ⤵ | SLP (12) ⤴ | BFI (9) ⤴ | TM (9) ~ | TMI (8) ⤵ |
| *Cottus carolinae* | NR | TM (22) ⤵ | TMI (17) ⤵ | PPT (17) ⤵ | POP (12) ⤴ | C_WA (7) ~ |
| *Cottus confusus* | SN | D_AG (30) ⤵ | FHS (20) ⤴ | TM (12) ⤵ | MVU (10) ⤴ | C_GR (9) ~ |
| *Cyprinella galactura* | NR | SINU (22) ⤴ | PPT (19) ⤵ | TMI (11) ⤴ | TM (8) ~ | C_AG (6) ⤵ |
| *Cyprinella lutrensis* | IL | TMA (26) ⤴ | TM (22) ⤴ | SO (4) ⤴ | POP (4) ~ | BFI (4) ⤵ |
| *Cyprinella venusta* | BR | BFI (32) ⤴ | PPT (12) ~ | C_UB (5) ~ | C_WA (4) ⤴ | DRA (4) ⤴ |
| *Dorosoma petenense* | BR | C_WA (17) ⤴ | MFU (16) ⤴ | DRA (11) ⤴ | BFI (8) ⤵ | FHS (6) ⤵ |
| *Etheostoma blennioides* | NR | BFI (32) ⤵ | MVU (10) ⤴ | ELE (8) ⤵ | NT (8) ⤵ | C_WA (5) ⤴ |
| *Etheostoma caeruleum* | NR | SLP (19) ⤴ | MFU (17) ⤴ | TMA (9) ⤴ | ELE (8) ⤵ | PPT (8) ⤵ |
| *Etheostoma exile* | IL | BFI (21) ⤴ | D_AG (15) ⤵ | POP (11) ⤴ | PPT (10) ⤵ | RDX (8) ⤴ |
| *Etheostoma nigrum* | IL | ELE (29) ⤴ | NT (16) ~ | PT (11) ~ | BFI (9) ⤴ | POP (5) ~ |
| *Fundulus notatus* | IL | SLP (24) ⤵ | TMA (12) ⤴ | TMI (11) ⤴ | D_FR (8) ⤵ | SINU (6) ⤴ |
| *Gambusia affinis* | BR | C_FR (19) ⤵ | C_AG (18) ~ | MVU (14) ⤴ | ELE (7) ⤴ | NT (7) ⤴ |
| *Hypentelium nigricans* | NR | BFI (31) ⤵ | NT (14) ⤴ | ELE (12) ⤵ | MVU (8) ⤴ | PT (5) ⤴ |
| *Ictalurus punctatus* | BR | SLP (14) ⤵ | C_AG (9) ⤴ | PPT (8) ~ | SO (7) ⤴ | MVU (6) ~ |
| *LepiSOsteus osseus* | BR | POP (17) ⤴ | SLP (13) ⤵ | C_WA (10) ⤴ | ELE (8) ⤵ | FHS (8) ⤵ |
| *Lepomis humilis* | BR | C_AG (23) ⤵ | ELE (15) ~ | C_WA (12) ⤴ | BFI (9) ⤵ | RDX (7) ⤵ |
| *Lepomis megalotis* | BR | PPT (14) ⤴ | D_FR (13) ~ | C_WA (9) ⤴ | BFI (9) ⤴ | TMI (8) ⤴ |
| *Luxilus chrysocephalus* | IL | FHS (12) ⤵ | BFI (11) ~ | D_FR (8) ⤵ | D_AG (8) ⤴ | TMA (7) ~ |
| *Luxilus chrysocephalus* | NR | TMI (40) ⤵ | TMA (9) ⤴ | NT (6) ⤴ | BFI (6) ⤵ | POP (6) ⤴ |
| *Luxilus cornutus* | IL | TM (38) ⤵ | TMI (14) ⤵ | SINU (7) ⤵ | C_WA (5) ~ | D_AG (4) ⤴ |
| *Menidia beryllina* | BR | SO (21) ⤴ | C_WA (19) ⤴ | MVU (15) ⤴ | MFU (5) ⤴ | DRA (5) ⤴ |
| *Nocomis biguttatus* | IL | D_FR (15) ⤵ | D_AG (10) ⤴ | FHS (9) ⤵ | MVU (9) ⤴ | SO (8) ⤵ |
| *Nocomis leptocephalus* | NR | BFI (17) ⤴ | D_FR (10) ⤵ | SO (7) ~ | MVU (7) ⤴ | RDX (4) ~ |
| *Notropis atherinoides* | IL | ELE (29) ⤵ | D_AG (13) ⤵ | MVU (5) ⤴ | C_AG (5) ⤵ | TMA (4) ⤵ |
| *Notropis dorsalis* | IL | TM (14) ⤴ | POP (9) ~ | TMA (7) ⤴ | PPT (6) ~ | D_AG (6) ⤴ |
| *Notropis hudsonius* | IL | ELE (12) ⤵ | DRA (10) ⤴ | POP (7) ~ | C_AG (7) ⤵ | RDX (6) ~ |
| *Notropis rubellus* | NR | BFI (30) ⤵ | POP (8) ⤵ | MVU (8) ⤴ | TMI (7) ⤵ | PPT (6) ~ |
| *Notropis stramineus* | IL | C_UB (22) ⤵ | D_AG (19) ⤴ | FHS (9) ⤴ | C_FR (8) ⤵ | SINU (5) ⤴ |
| *Notropis volucellus* | NR | ELE (20) ⤵ | C_WA (17) ⤴ | POP (11) ⤵ | TMA (6) ~ | MFU (5) ⤴ |
| *Noturus gyrinus* | BR | ELE (27) ⤵ | TMI (14) ⤴ | BFI (13) ⤵ | TM (7) ⤴ | D_FR (6) ⤵ |
| *Pimephales notatus* | NR | BFI (50) ⤵ | ELE (8) ⤵ | PPT (6) ⤵ | TMI (3) ⤵ | POP (3) ⤴ |
| *Pimephales promelas* | BR | TMA (19) ⤴ | DRA (13) ⤵ | RDX (12) ⤴ | BFI (6) ⤵ | POP (6) ⤴ |
| *Pimephales vigilax* | BR | MVU (14) ⤴ | TMA (9) ⤴ | SINU (8) ⤴ | MFU (7) ⤴ | FHS (6) ~ |
| *ProSOpium williamsoni* | SN | D_AG (20) ⤵ | C_UB (15) ⤴ | SINU (10) ⤴ | C_FR (10) ⤵ | MVU (7) ⤴ |
| *Ptychocheilus oregonensis* | SN | BFI (45) ⤵ | C_AG (9) ⤴ | TMI (6) ⤴ | TM (5) ⤴ | MVU (5) ⤵ |
| *Rhinichthys cataractae* | SN | SINU (39) ⤴ | C_AG (9) ⤵ | BFI (9) ⤴ | DRA (6) ~ | RDX (5) ⤴ |
| *Rhinichthys cataractae* | NR | BFI (32) ⤵ | RDX (20) ⤵ | D_AG (8) ~ | TM (5) ⤵ | MVU (4) ⤴ |
| *Richardsonius balteatus* | SN | DRA (17) ⤴ | C_GR (10) ⤴ | FHS (9) ⤵ | BFI (9) ⤵ | C_FR (7) ⤵ |
| *Amia calva* | IL | TMA (35) ~ | TM (13) ~ | FHS (11) ⤴ | BFI (8) ⤴ | ELE (6) ~ |
| *Etheostoma microperca* | IL | BFI (20) ⤴ | NT (13) ⤴ | RDX (13) ⤴ | TMA (7) ⤵ | FHS (6) ⤵ |
| *Lythrurus ardens* | NR | PPT (18) ⤵ | SINU (11) ⤴ | BFI (9) ~ | TMA (8) ~ | ELE (8) ⤵ |
| *Notropis buccatus* | IL | ELE (19) ⤵ | TMI (10) ~ | POP (9) ~ | NT (9) ⤴ | TMA (5) ⤵ |
| *Notropis buccatus* | NR | BFI (25) ⤵ | ELE (12) ⤵ | SLP (8) ~ | C_AG (8) ⤵ | C_FR (6) ⤴ |
| *Opsopoeodus emiliae* | IL | ELE (25) ~ | BFI (9) ⤴ | MVU (9) ⤴ | DRA (7) ⤴ | POP (6) ~ |
| *Opsopoeodus emiliae* | BR | TMI (30) ⤴ | TM (19) ⤴ | FHS (8) ⤵ | D_AG (6) ~ | ELE (6) ⤵ |
| *Campostoma oligolepis* | IL | TMI (16) ~ | TM (11) ~ | SINU (8) ⤴ | NT (7) ⤴ | POP (6) ~ |
| *Carpiodes velifer* | IL | TMI (17) ~ | TMA (16) ⤴ | SINU (11) ⤵ | C_UB (8) ⤴ | MVU (7) ⤴ |
| *Cottus beldingii* | SN | PPT (30) ⤴ | SLP (18) ⤴ | D_UB (10) ~ | RDX (9) ⤵ | MFU (3) ⤴ |
| *Cottus rhotheus* | SN | C_UB (36) ⤵ | D_AG (18) ⤵ | DRA (12) ⤵ | PPT (9) ~ | POP (5) ⤵ |
| *Cyprinella spiloptera* | IL | TM (10) ⤵ | TMA (9) ⤵ | SINU (9) ~ | MVU (7) ⤴ | PPT (7) ⤵ |
| *Cyprinella spiloptera* | NR | ELE (17) ⤵ | MFU (16) ⤴ | RDX (8) ⤴ | POP (7) ⤵ | BFI (7) ~ |
| *Etheostoma chlorosoma* | IL | C_WA (14) ⤴ | ELE (14) ⤵ | MVU (8) ⤵ | D_AG (7) ⤵ | TM (7) ⤵ |
| *Etheostoma chlorosoma* | BR | PPT (43) ⤴ | C_AG (6) ⤵ | TMA (6) ⤵ | D_FR (6) ⤴ | POP (4) ⤵ |
| *Etheostoma spectabile* | IL | MVU (9) ⤵ | BFI (9) ⤵ | POP (8) ~ | SLP (7) ⤴ | TMI (6) ⤵ |
| *Etheostoma spectabile* | BR | BFI (42) ⤴ | TMA (7) ⤴ | SLP (7) ⤴ | C_UB (5) ⤴ | PPT (5) ~ |
| *Hybognathus nuchalis* | IL | ELE (17) ⤵ | TMA (12) ⤴ | TM (11) ⤴ | DRA (10) ⤴ | MVU (8) ⤴ |
| *Ictiobus bubalus* | IL | ELE (29) ⤵ | C_WA (10) ⤴ | DRA (6) ⤴ | NT (6) ⤴ | MFU (5) ⤴ |
| *Notropis buchanani* | BR | MVU (15) ~ | MFU (15) ⤴ | C_WA (10) ⤵ | BFI (8) ⤵ | SO (6) ⤵ |
| *Percina phoxocephala* | IL | TMA (19) ⤴ | SINU (9) ⤴ | SLP (8) ~ | C_FR (7) ⤵ | D_AG (6) ⤴ |
| *Chrosomus erythrogaster* | IL | TMI (14) ⤵ | NT (11) ⤵ | RDX (10) ⤴ | TM (9) ⤵ | TMA (7) ⤴ |
| *Etheostoma asprigene* | IL | ELE (33) ⤵ | MVU (7) ⤴ | C_WA (5) ~ | D_FR (4) ~ | D_AG (4) ⤵ |
| *Etheostoma gracile* | BR | PPT (39) ⤴ | TMI (16) ⤴ | D_AG (6) ~ | POP (5) ⤵ | BFI (4) ⤵ |
| *Percina sciera* | BR | C_FR (11) ~ | MVU (11) ⤴ | C_UB (10) ~ | SINU (9) ⤴ | PPT (7) ⤴ |
| *Catostomus ardens* | SN | C_AG (33) ⤴ | BFI (27) ~ | POP (8) ⤴ | C_UB (6) ⤴ | TMI (6) ⤵ |
| *Luxilus cerasinus* | NR | BFI (17) ~ | TMA (13) ~ | D_AG (10) ⤴ | TM (9) ⤵ | C_UB (8) ⤵ |
| *Nocomis platyrhynchus* | NR | BFI (17) ⤵ | MVU (10) ~ | TMI (6) ⤵ | PPT (6) ~ | SO (6) ⤴ |
| *Notropis rubricroceus* | NR | SINU (22) ⤵ | BFI (14) ⤴ | D_AG (8) ⤴ | C_UB (6) ⤵ | RDX (6) ⤵ |
| *Percina oxyrhynchus* | NR | MVU (20) ⤴ | MFU (11) ⤴ | ELE (7) ⤵ | SINU (5) ⤵ | DRA (5) ⤴ |
| *Percina roanoka* | NR | MVU (15) ⤴ | DRA (10) ⤴ | BFI (9) ⤴ | ELE (9) ~ | TMI (6) ⤴ |
| *Chrosomus oreas* | NR | D_FR (12) ⤵ | TMI (11) ~ | C_FR (8) ⤵ | D_AG (7) ⤴ | SO (6) ~ |
| *Etheostoma kanawhae* | NR | BFI (28) ⤴ | SO (12) ~ | SLP (6) ~ | SINU (6) ⤴ | MVU (5) ⤵ |
| *Etheostoma osburni* | NR | TMI (17) ⤵ | D_FR (9) ⤴ | FHS (8) ⤴ | BFI (7) ⤵ | TM (6) ⤵ |
| *Notropis scabriceps* | NR | TMI (41) ⤵ | BFI (21) ⤴ | PPT (4) ⤴ | TM (4) ⤵ | SLP (4) ⤴ |
| *Luxilus albeolus* | NR | TMA (15) ⤴ | MFU (14) ⤴ | TM (12) ⤴ | DRA (9) ⤴ | C_UB (6) ~ |
| *Exoglossum laurae* | NR | TM (13) ⤵ | C_WA (9) ⤵ | TMA (7) ~ | NT (6) ⤴ | TMI (6) ⤵ |
| *Phenacobius teretulus* | NR | BFI (35) ⤴ | C_AG (10) ⤴ | TMI (5) ⤴ | C_FR (5) ~ | TM (5) ⤵ |

**Figure A.** **An illustration of inferring absences based on historical fish presence records.** Each row of Matrix A represents a presence record of a fish species in a unit. Units in Matrix A can be NHD (National Hydrography Dataset) inter-confluence stream segments, HUC-8 or HUC-12 watersheds. We use Type N and G to distinguish non-game species and game species. The 1’s in the Matrix B are fish presences, and 0’s are fish absences inferred from presences of one or more other non-game species. In the example, only two game species (S3 and S6) are found at unit U4, which are considered non-informative to infer absence of non-game species, so the cells at unit U4 for all non-game species (S1, S2, S4, S5, S7, and S8) are left blank.


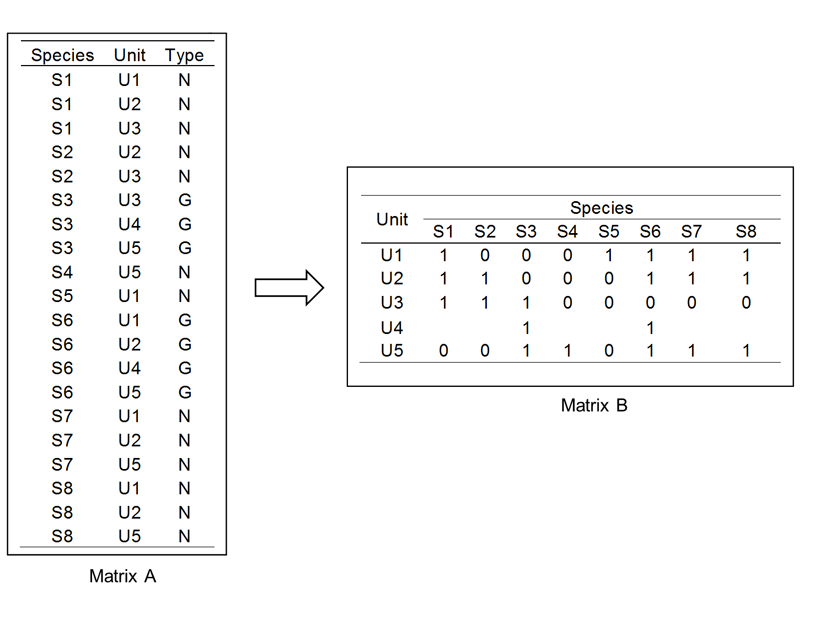


**Figure B. Relationship of model performance and species prevalence**. A figure showing the correlation of the model performance of boosted regression tree models in terms of AUC (the area under the receiver-operating-characteristic curve) and the observed prevalence of stream fish species in the four selected basins (i.e., New River, Illinois River, Brazos River, and Snake River). The observed prevalence is the proportion of presence observations in all observations. This nonlinear negative correlation suggests that the habitat requirements and spatial distributions of more common species tend to be difficult to model.


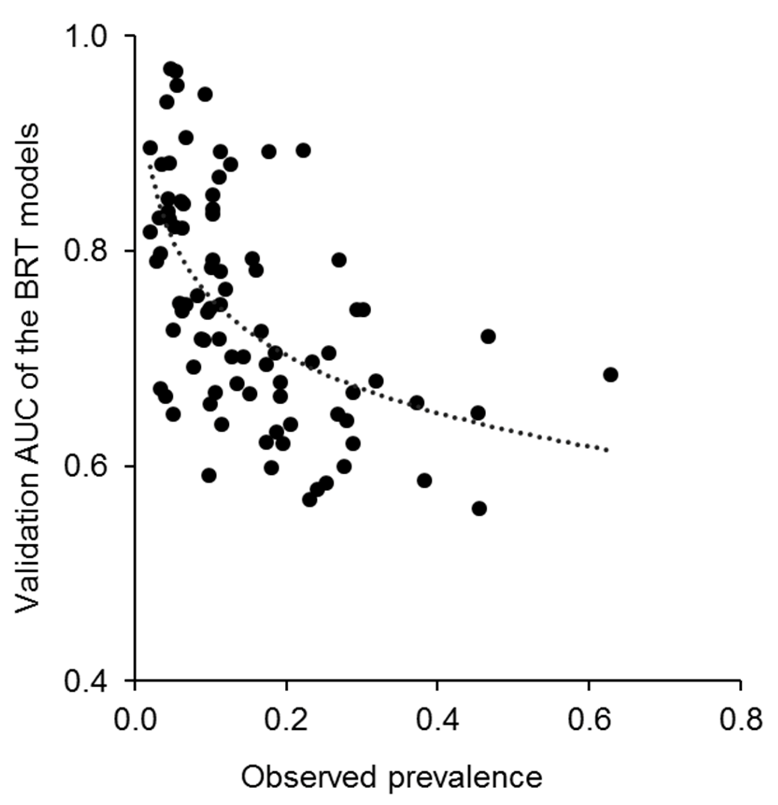

Supplement: S3 File — A table listing the fish species modeled in this study (Table A). Summary of performance (in terms of AUC) of logistic models with Lasso regularization (LM) and boosted regression tree (BRT) models in the training process (_train) and cross validation (_cv) for the 76 fish species in four river basins (BR-Brazos River, IL-Illinois River, NR-New River, SN-Snake River) (Table B). A summary on the key habitat factors for each of the 76 stream fish species in four river basins (i.e., BR-Brazos River, IL-Illinois River, NR-New River, SN-Snake River) in the non-spatial boosted regression tree (BRT) models (Table C). An illustration of inferring absences based on historical fish presence records (Figure A). Relationship of model performance and species prevalence (Figure B). (DOCX) [file pone.0129995.s003.docx]
